# Supplementary material for: Aging impairs the antiviral defense in Caenorhabditis elegans due to loss of DRH-1/RIG-I deSUMOylation by ULP-4/SENP7
Source: EMBO Rep. 2025 Oct 2;26(22):5459–82. doi: 10.1038/s44319-025-00589-0 (PMC12635358; doi:10.1038/s44319-025-00589-0)
Supplement: Supplementary file 1 — Appendix [file 44319_2025_589_MOESM1_ESM.pdf]

Table of Contents

Appendix Figures S1-S5& Figure legends

Appendix Figure S1: ULP-4 is not required for induction of the IPR by non-viral triggers. pg. 2
Appendix Figure S2. Viral infection does not change DRH-1 protein levels. pg. 3
Appendix Figure S3. SMO-1 overlaps with DRH-1 under uninfected condition. pg. 4
Appendix Figure S4. K647/K731 are the predicted target residues for DRH-1 SUMOylation. pg. 5
Appendix Figure S5. nonSUMOylated DRH-1 co-localizes with mitochondria. pg. 6

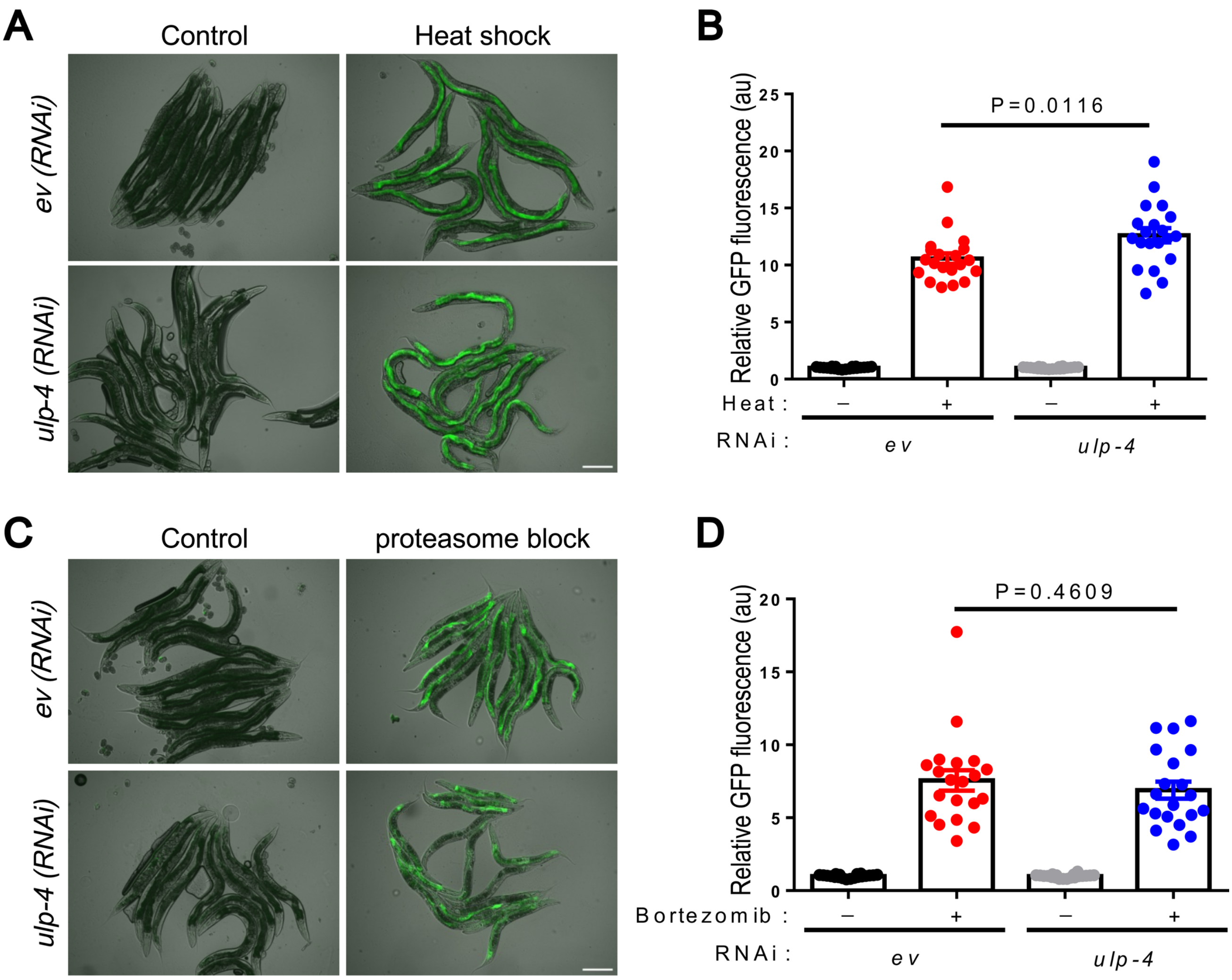

Appendix Figure S1. ULP-4 is not required for induction of the IPR by non-viral triggers. (*A*) Representative images of *pals-5p::GFP* expression after empty vector or *ulp-4(RNAi)* treatment, followed by prolonged 28ºC heat stress ("Heat Shock"). **(*B*)** Quantification of GFP fluorescence in (*A*). **(*C*)** Representative images of *pals-5p::GFP* expression after empty vector or *ulp-4(RNAi)* treatment, followed by bortezomib treatment. **(*D*)** Quantification of GFP fluorescence in (*C*). Scale bar = 200 µm. In all cases, values are the mean of 60 animals across three independent trials; error bars are the SEM. A two-tailed t test was used to calculate P-values, which is provided within the panel.

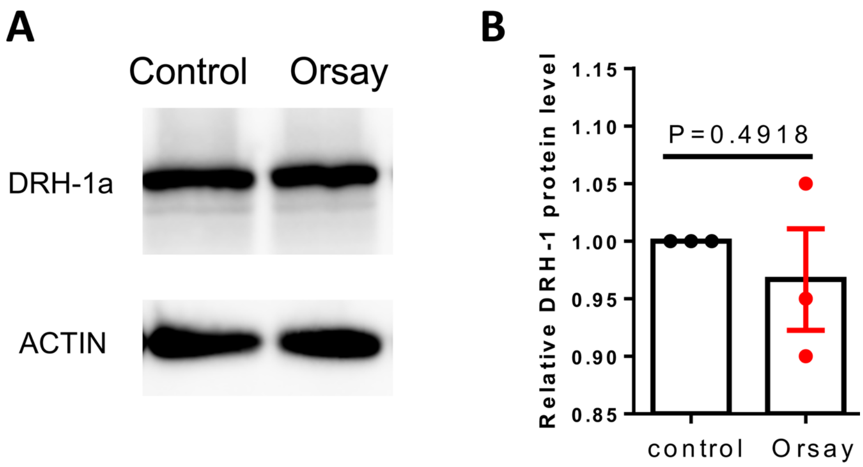

Appendix Figure S2. Viral infection does not change DRH-1 protein levels. (*A*) Representative immunoblot of mScarlet::DRH-1 in animals +/- viral treatment. **(*B*)** Quantification of DRH-1 levels. Values are the mean from three independent experimental replicates; error bars are the SEM. A two-tailed t test was used to calculate P-value, which is provided within the panel.

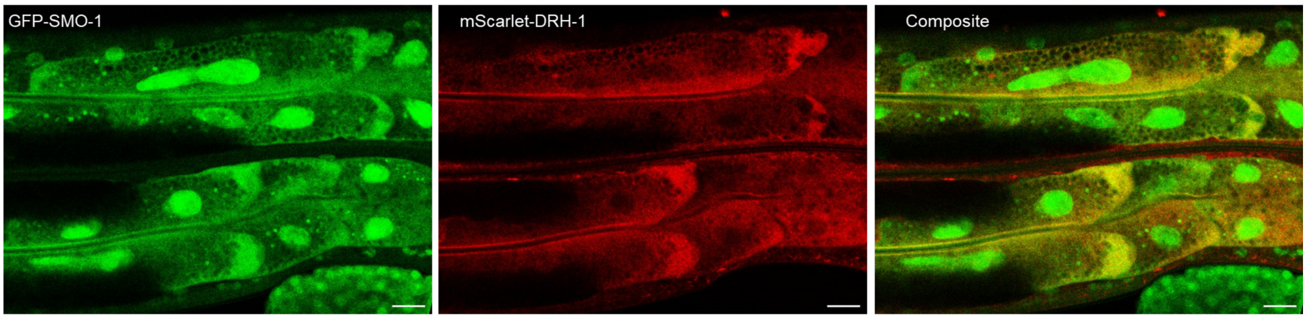

GFP-SMO-1;mScarlet-DRH-1

Appendix Figure S3. SMO-1 overlaps with DRH-1 under uninfected condition. Representative images of uninfected animals expressing mScarlet::DRH-1; GFP::SMO-1. Scale bar = 10 µm.

A

position	sequence	SUMOplot	JASSA	GPS-SUMO	MusiteDeep
K647	VALNY **LKDE** MEYRT	0.91	LOW	0.735	0.764
K731	LLMLG **IKSE** WMSGL	0.94	HIGH	0.826	0.926

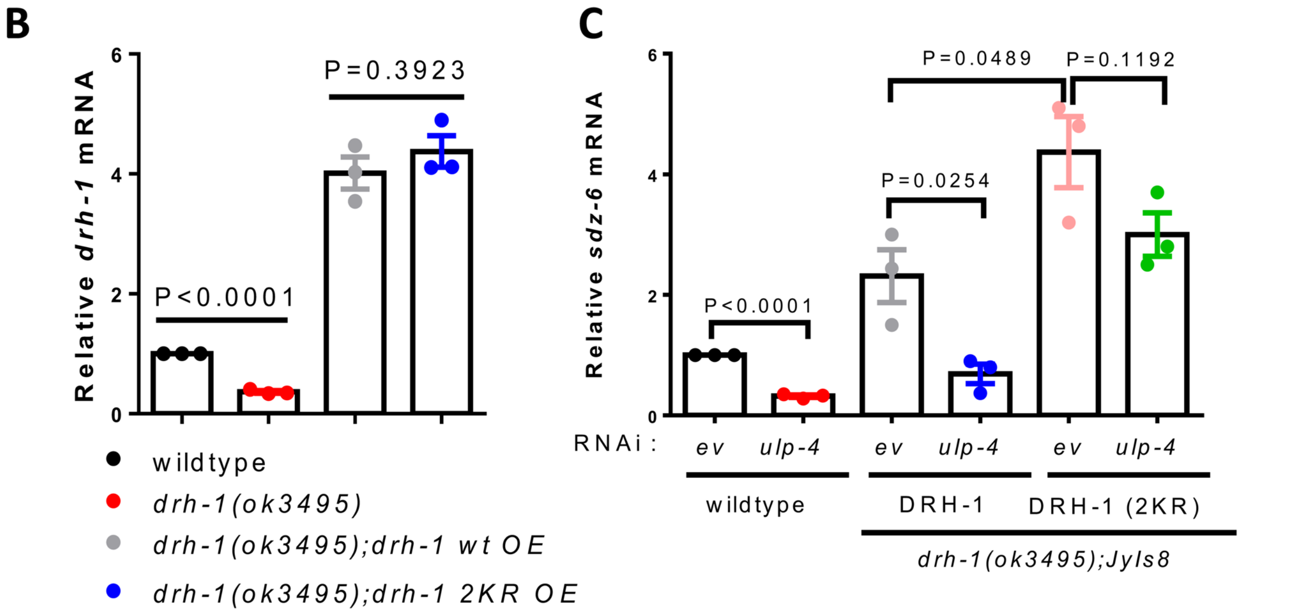

Appendix Figure S4. K647/K731 are the predicted target residues for DRH-1 SUMOylation. (***A***) SUMOylation likelihood scores for DRH-1 lysines 647 and 731 as predicted by four independent computational models. The detailed likelihood for all 60 DRH-1 lysines are listed in *Dataset S1*. (***B***) RT-qPCR analysis of endogenous *drh-1* in wildtype, *drh-1(ok3495)* null mutant, and *drh-1(ok3495)* null mutant animals with restored expression throughout the soma (*rpl-28p*) of either wildtype DRH-1 (*mScarlet::DRH-1(wt)*) or nonSUMOylatable DRH-1 (*mScarlet::DRH-1(2KR)*). (***C***) RT-qPCR analysis of endogenous *sdz-6* under the indicated conditions. In all cases, values are the mean of three independent trials; error bars are the SEM. A two-tailed t test was used to calculate P-values, which are provided within the panels.

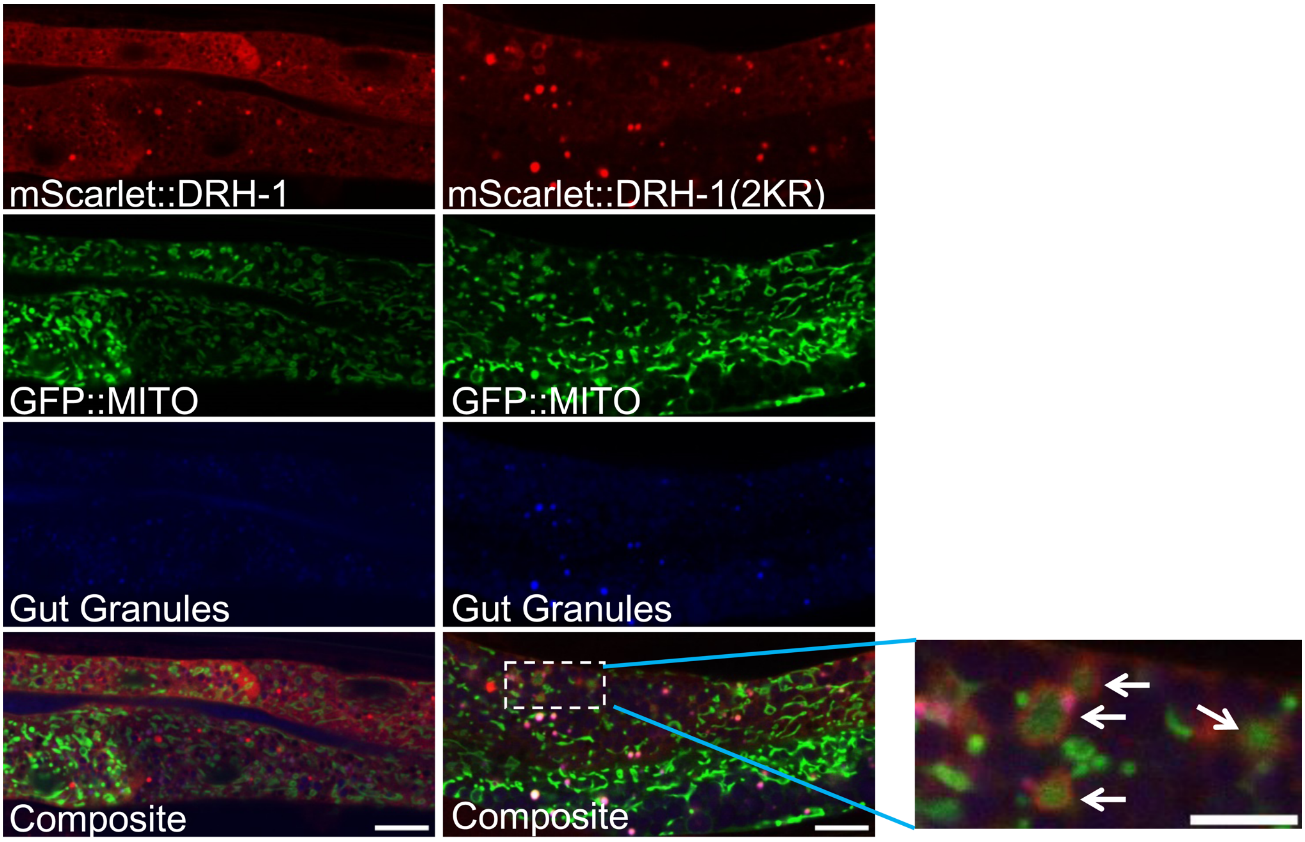

Appendix Figure S5. nonSUMOylated DRH-1 co-localizes with mitochondria. Representative images of uninfected animals expressing *mScarlet::DRH-1(WT); GFP::mito* or *mScarlet::DRH-1(2KR); GFP::mito* respectively, lysosomal related organelles/gut granules are indicated (405-nm blue channel). White arrows indicate DRH-1 localization to the outer membrane of the mitochondria. Composite images are reused in Figure 5E-G. Scale bar = 2 µm. Inset scale bar = 5 µm.
