# Supplementary figures and images for: Aging impairs the antiviral defense in Caenorhabditis elegans due to loss of DRH-1/RIG-I deSUMOylation by ULP-4/SENP7
Source: EMBO Rep. 2025 Oct 2;26(22):5459–82. doi: 10.1038/s44319-025-00589-0 (PMC12635358; doi:10.1038/s44319-025-00589-0)

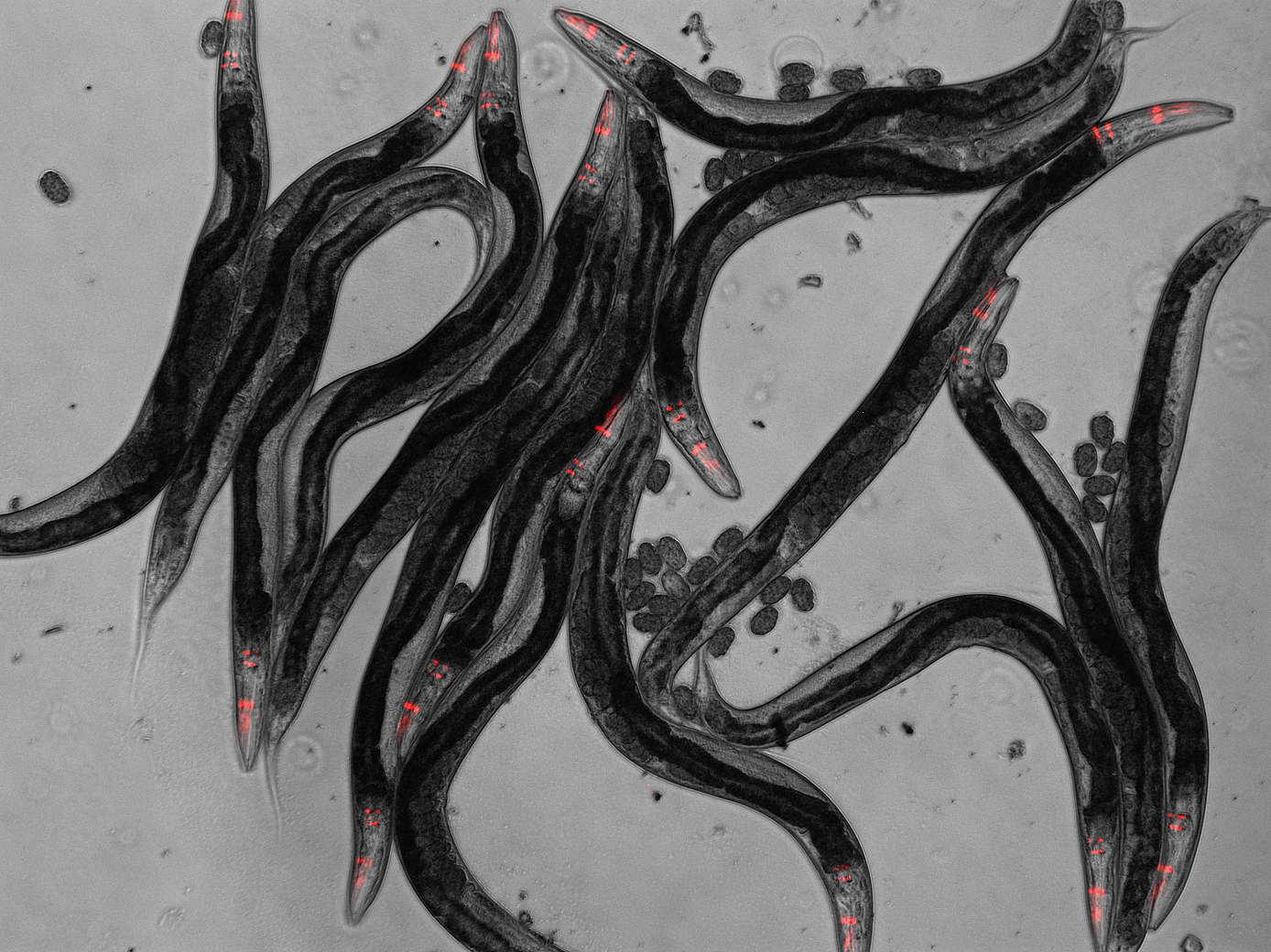

Supplement: Supplementary file 4 — Source data Fig. 1 [file 44319_2025_589_MOESM4_ESM.zip › EMBOR-2024-60913V2_Source-Data For Figure 1/1A/ev control.tif]

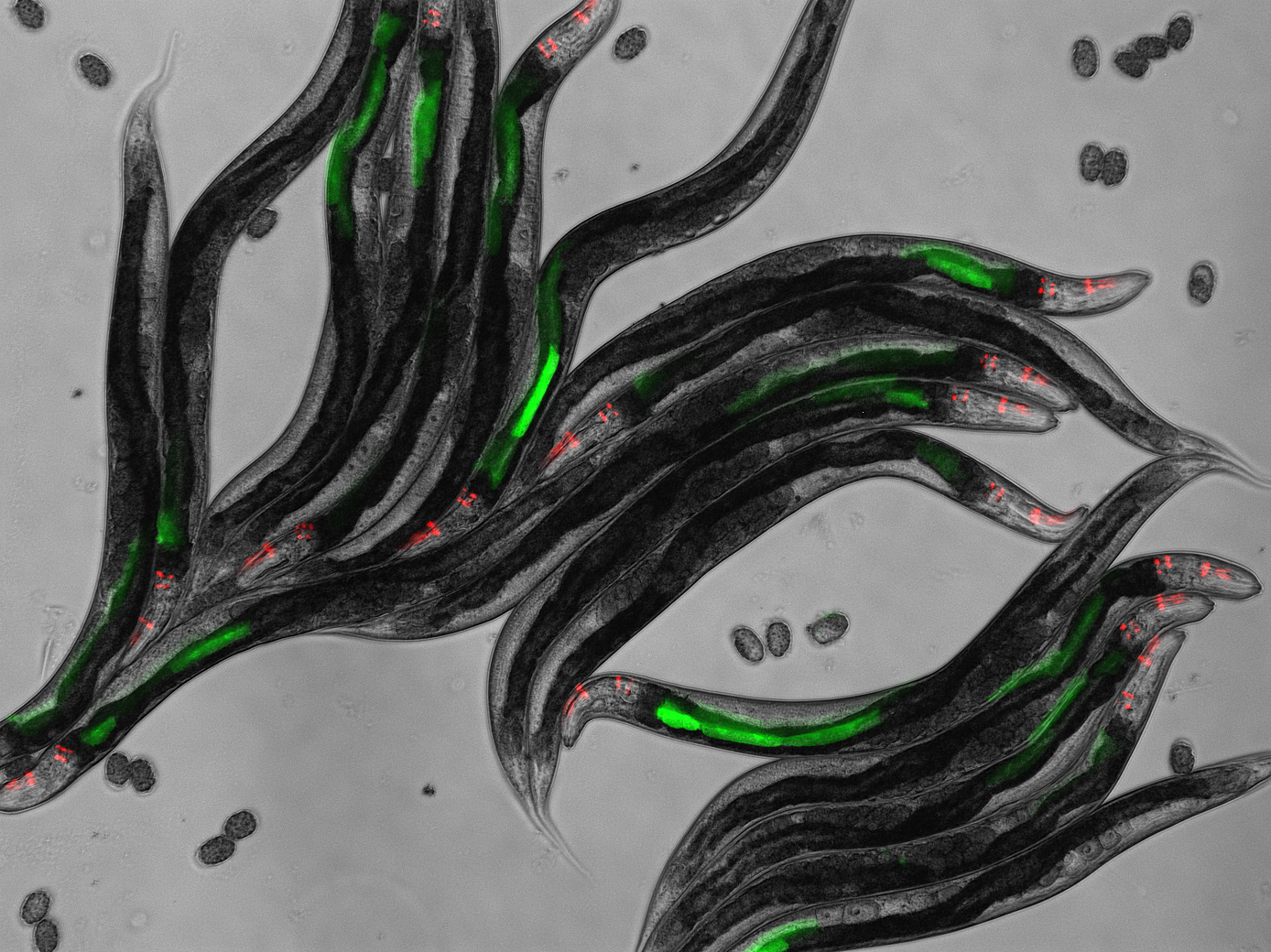

Supplement: Supplementary file 4 — Source data Fig. 1 [file 44319_2025_589_MOESM4_ESM.zip › EMBOR-2024-60913V2_Source-Data For Figure 1/1A/ev OV.tif]

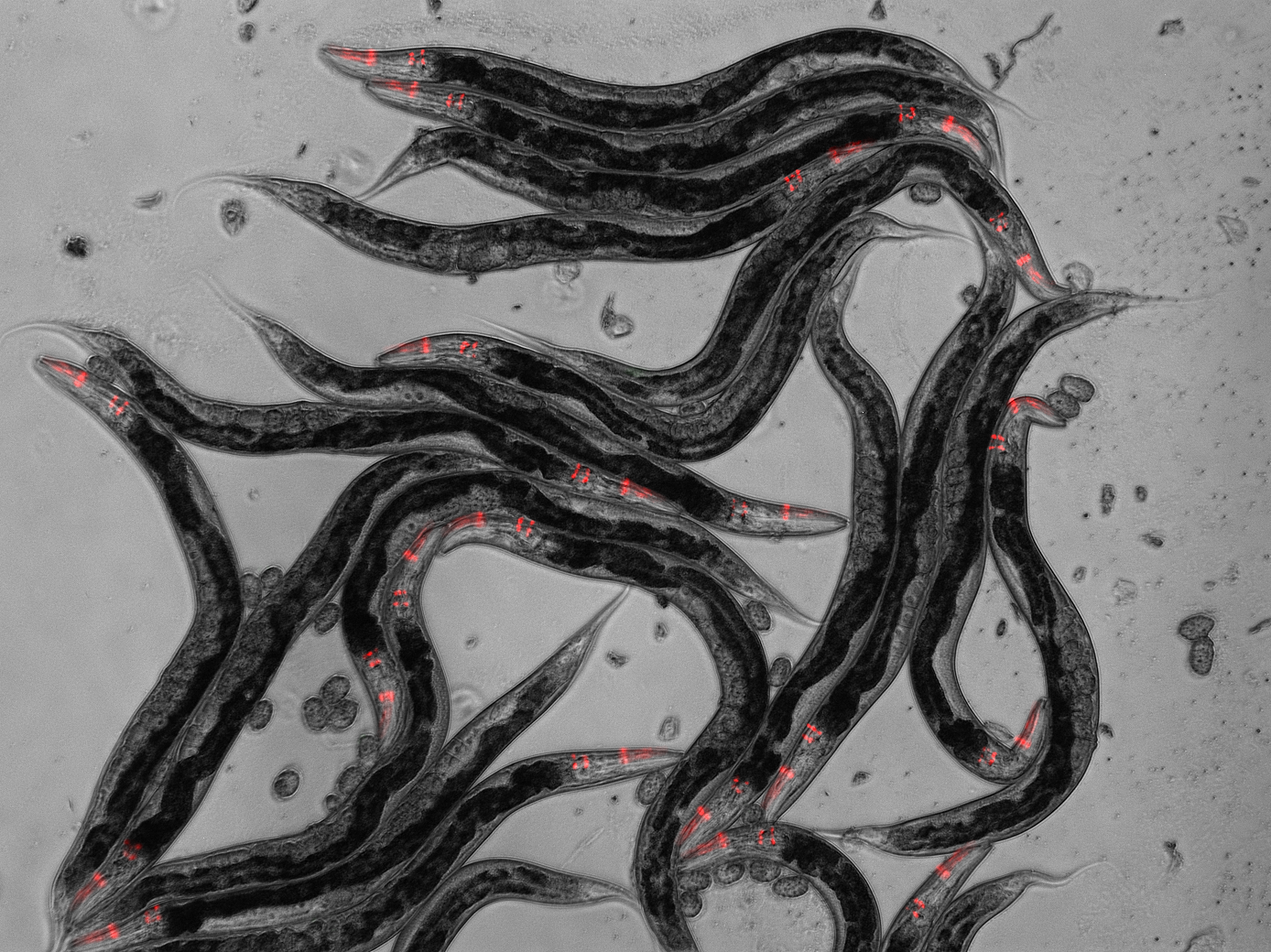

Supplement: Supplementary file 4 — Source data Fig. 1 [file 44319_2025_589_MOESM4_ESM.zip › EMBOR-2024-60913V2_Source-Data For Figure 1/1A/ulp-4 RNAi control.tif]

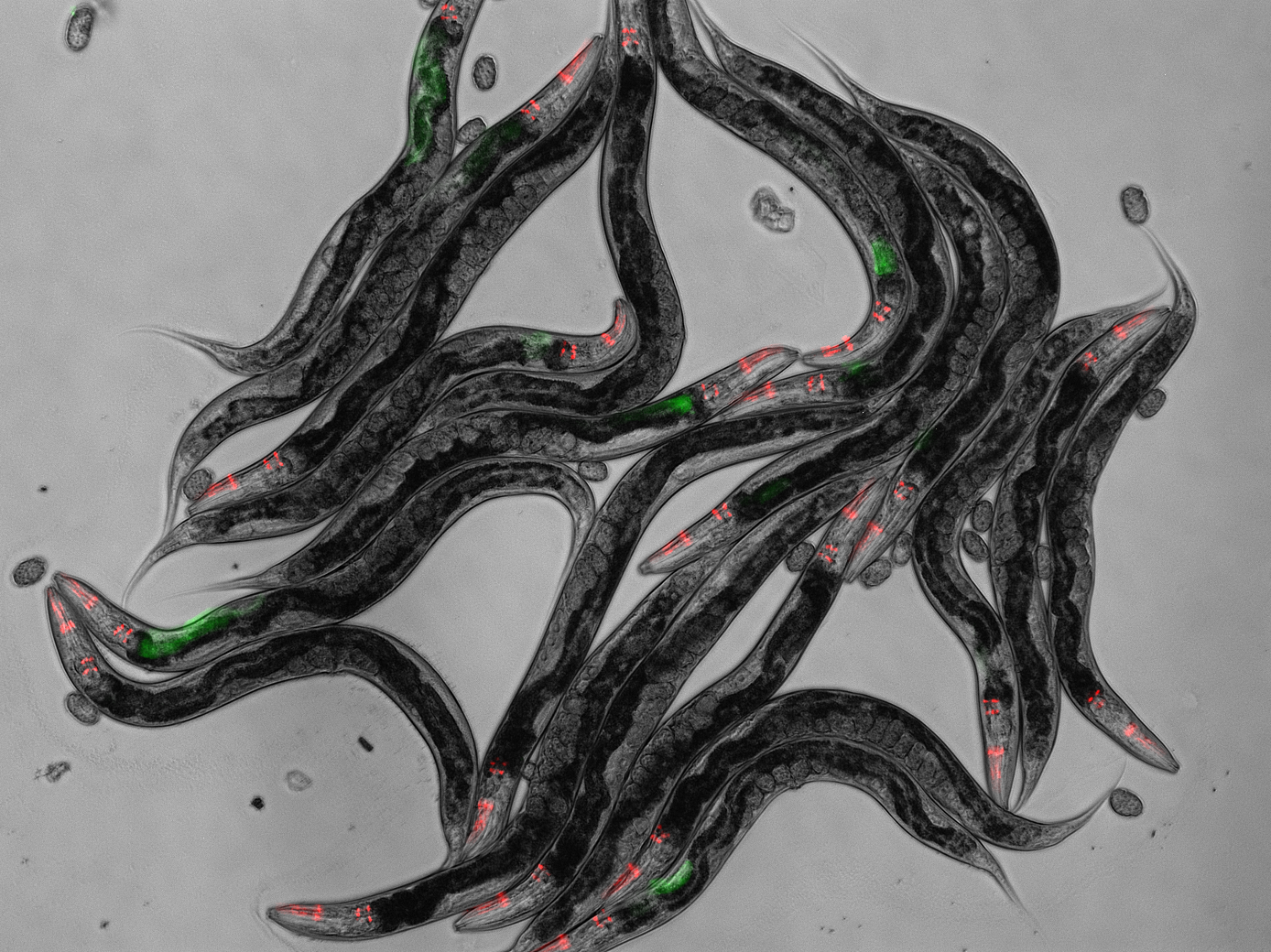

Supplement: Supplementary file 4 — Source data Fig. 1 [file 44319_2025_589_MOESM4_ESM.zip › EMBOR-2024-60913V2_Source-Data For Figure 1/1A/ulp-4 RNAi OV.tif]

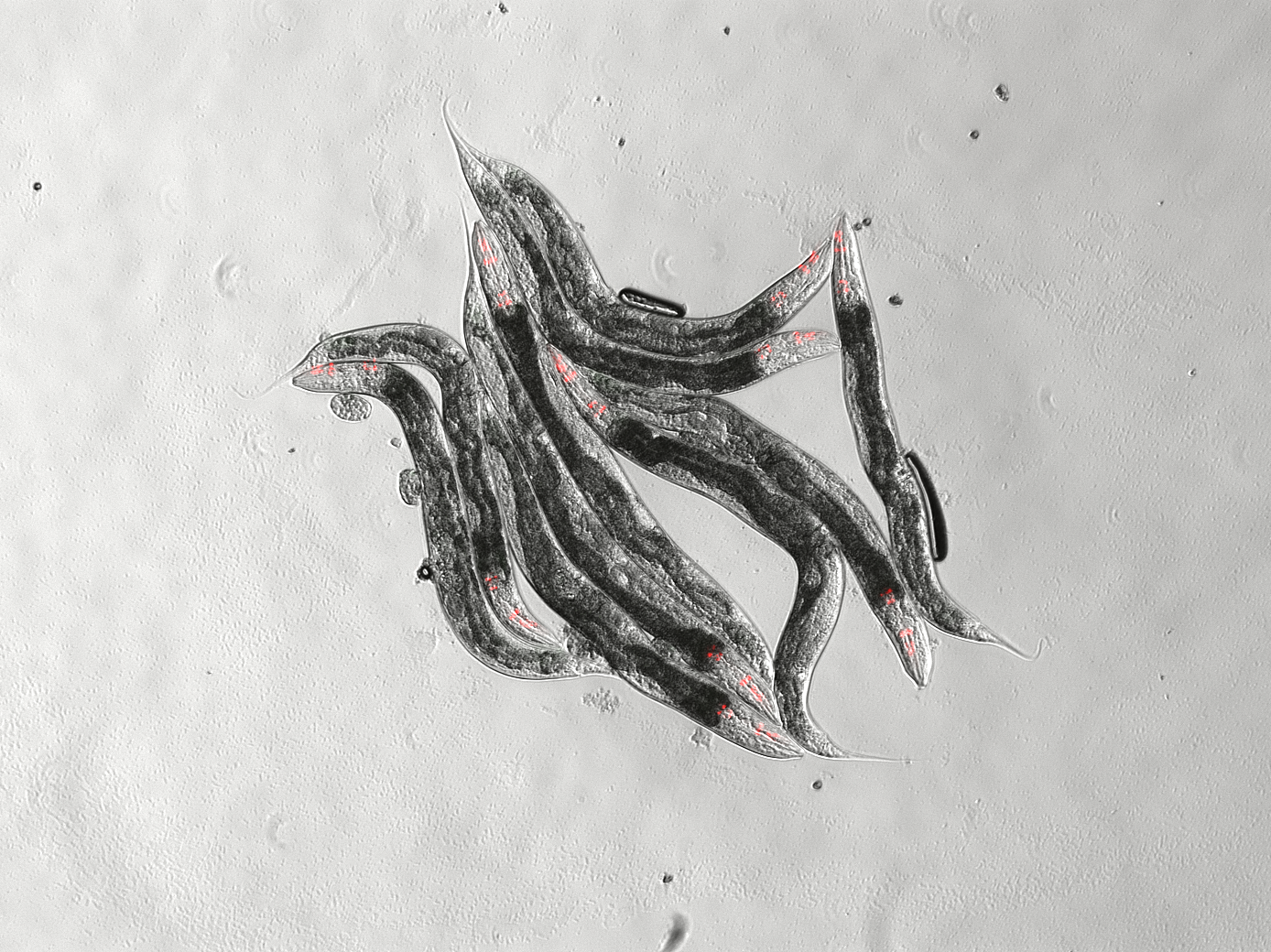

Supplement: Supplementary file 5 — Source data Fig. 2 [file 44319_2025_589_MOESM5_ESM.zip › EMBOR-2024-60913V3_Source-Data For Figure 2/2A/ulp-4(0) control.tif]

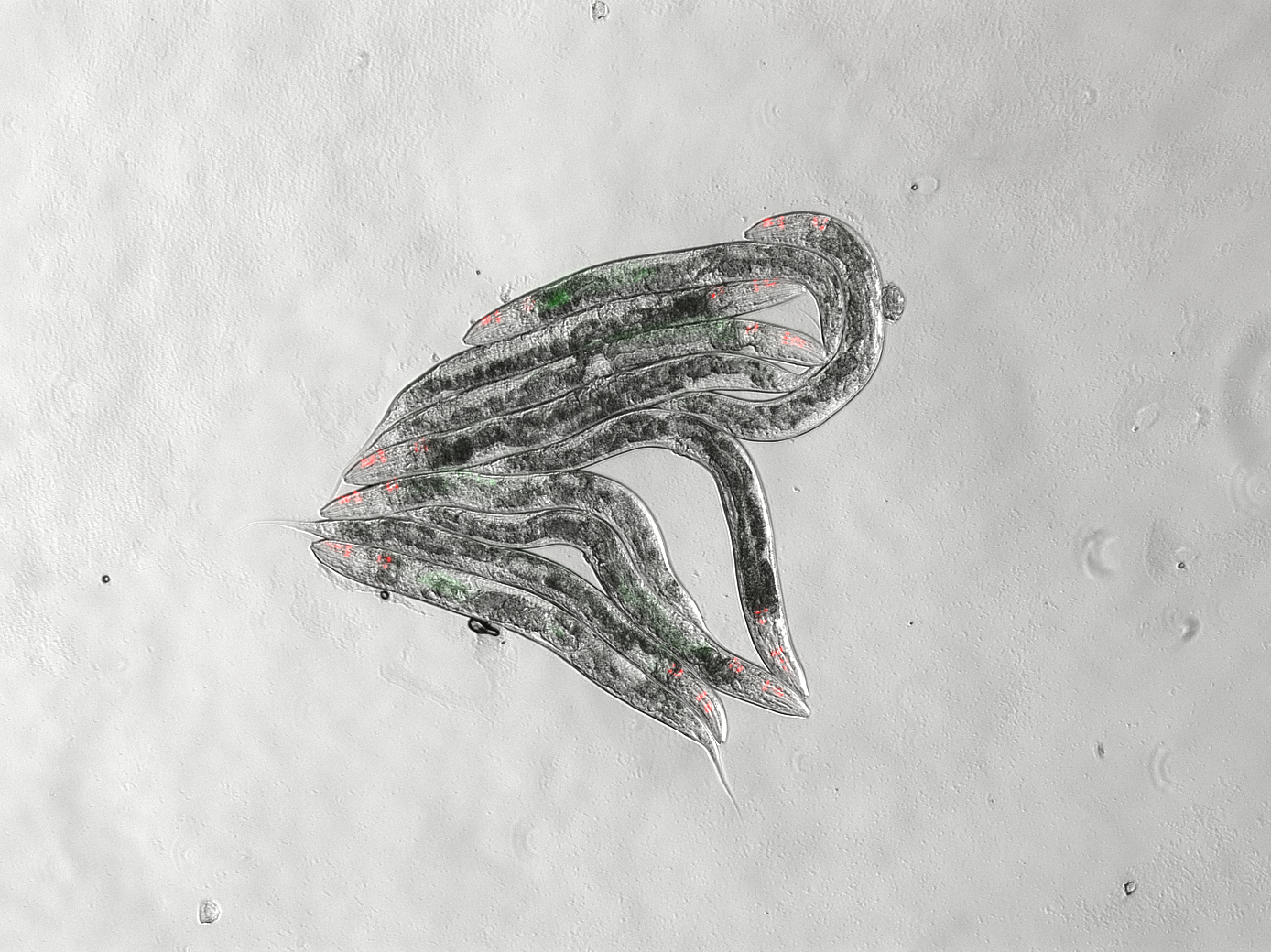

Supplement: Supplementary file 5 — Source data Fig. 2 [file 44319_2025_589_MOESM5_ESM.zip › EMBOR-2024-60913V3_Source-Data For Figure 2/2A/ulp-4(0) Orsay.tif]

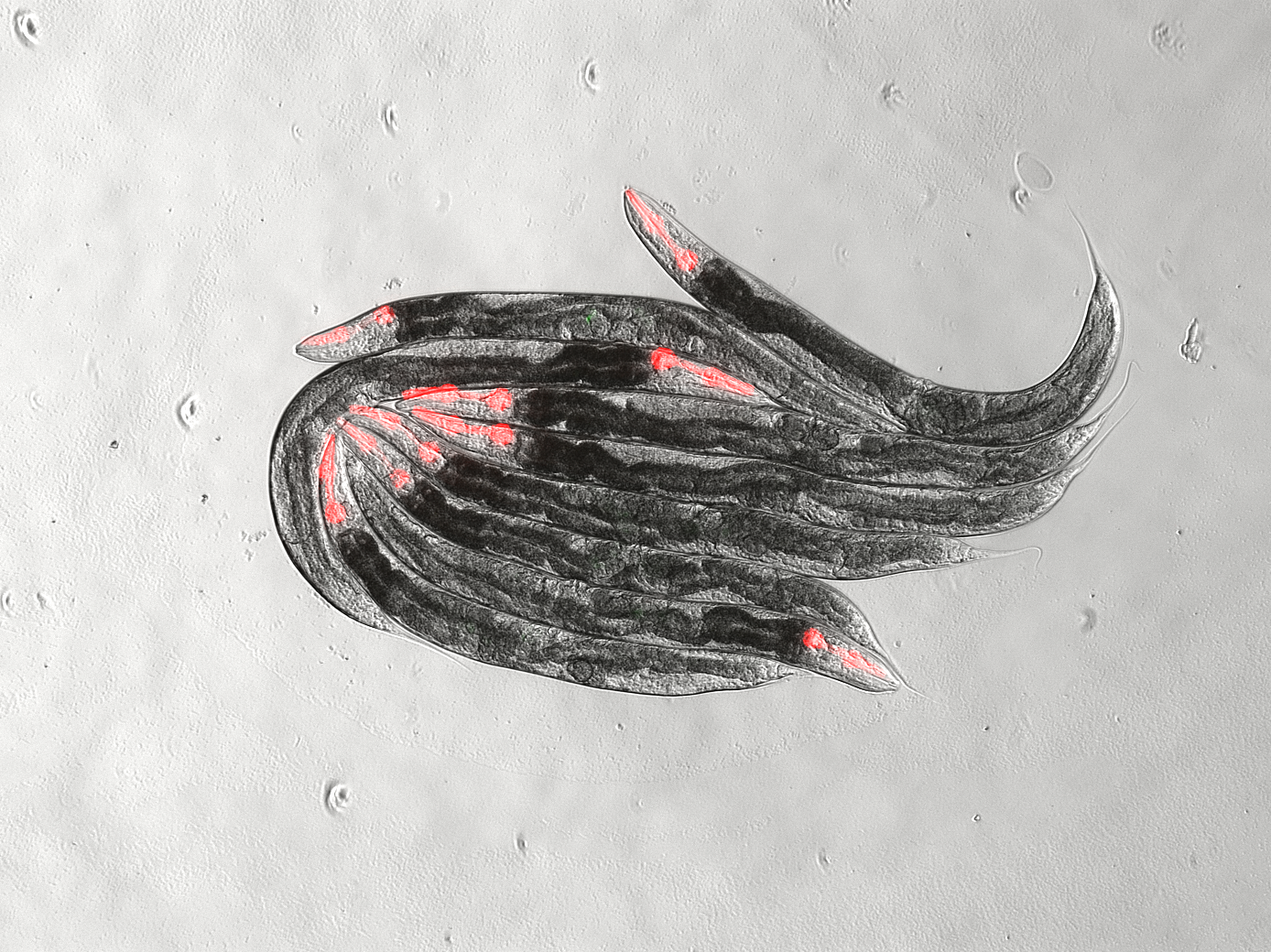

Supplement: Supplementary file 5 — Source data Fig. 2 [file 44319_2025_589_MOESM5_ESM.zip › EMBOR-2024-60913V3_Source-Data For Figure 2/2A/ulp-4(0), ulp-4 intestinal rescue control.tif]

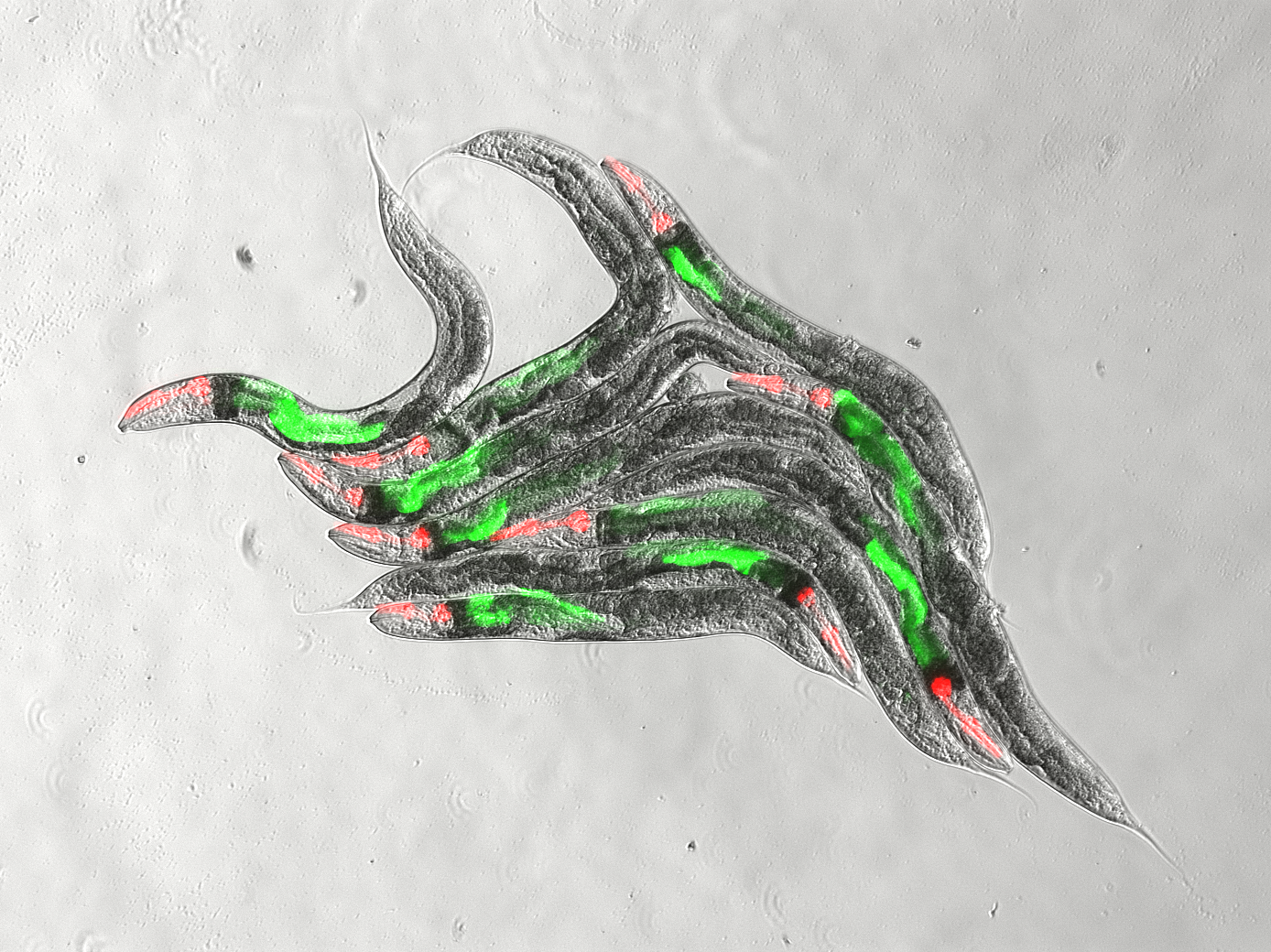

Supplement: Supplementary file 5 — Source data Fig. 2 [file 44319_2025_589_MOESM5_ESM.zip › EMBOR-2024-60913V3_Source-Data For Figure 2/2A/ulp-4(0), ulp-4 intestinal rescue Orsay.tif]

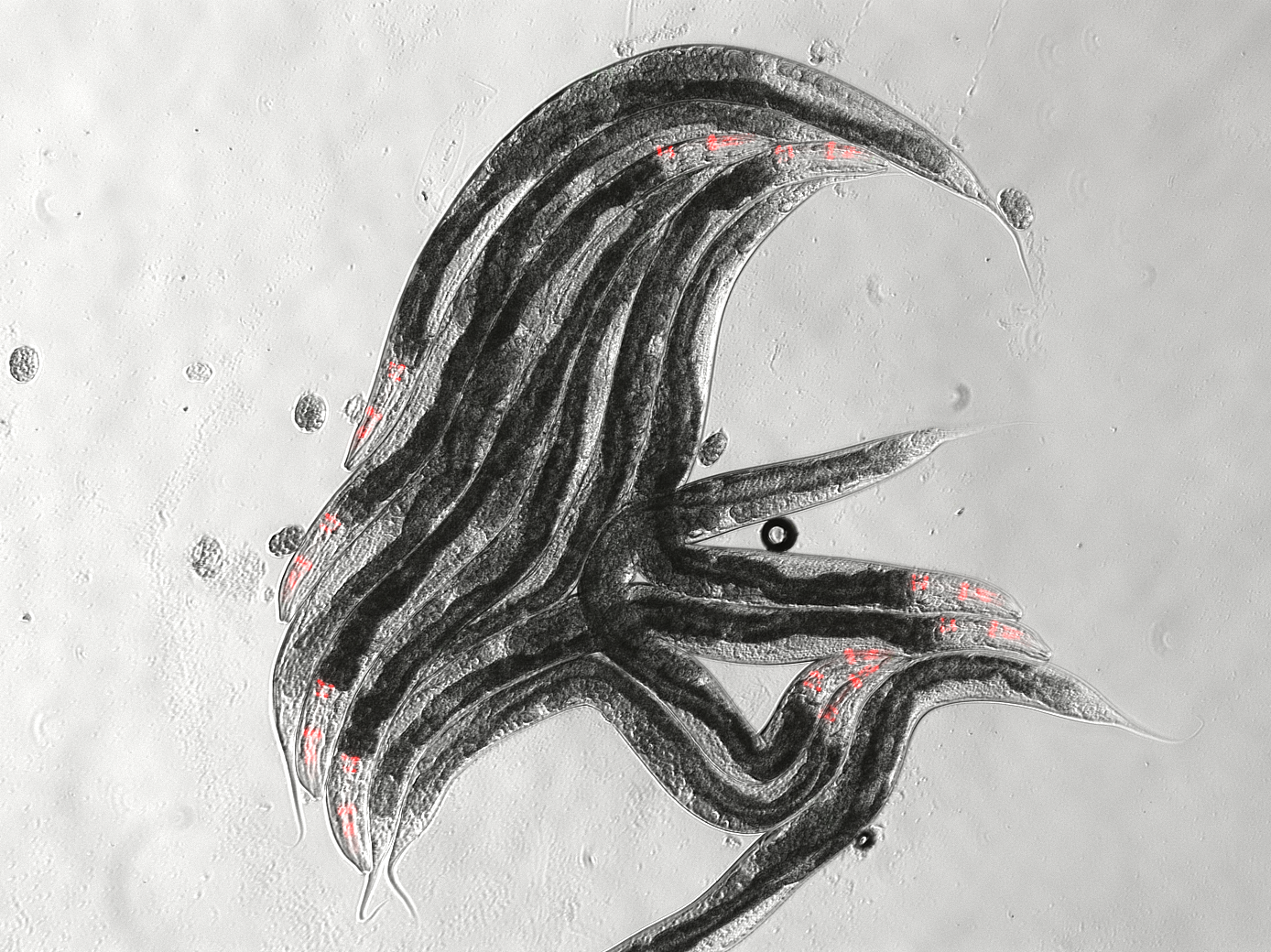

Supplement: Supplementary file 5 — Source data Fig. 2 [file 44319_2025_589_MOESM5_ESM.zip › EMBOR-2024-60913V3_Source-Data For Figure 2/2A/wildtype control.tif]

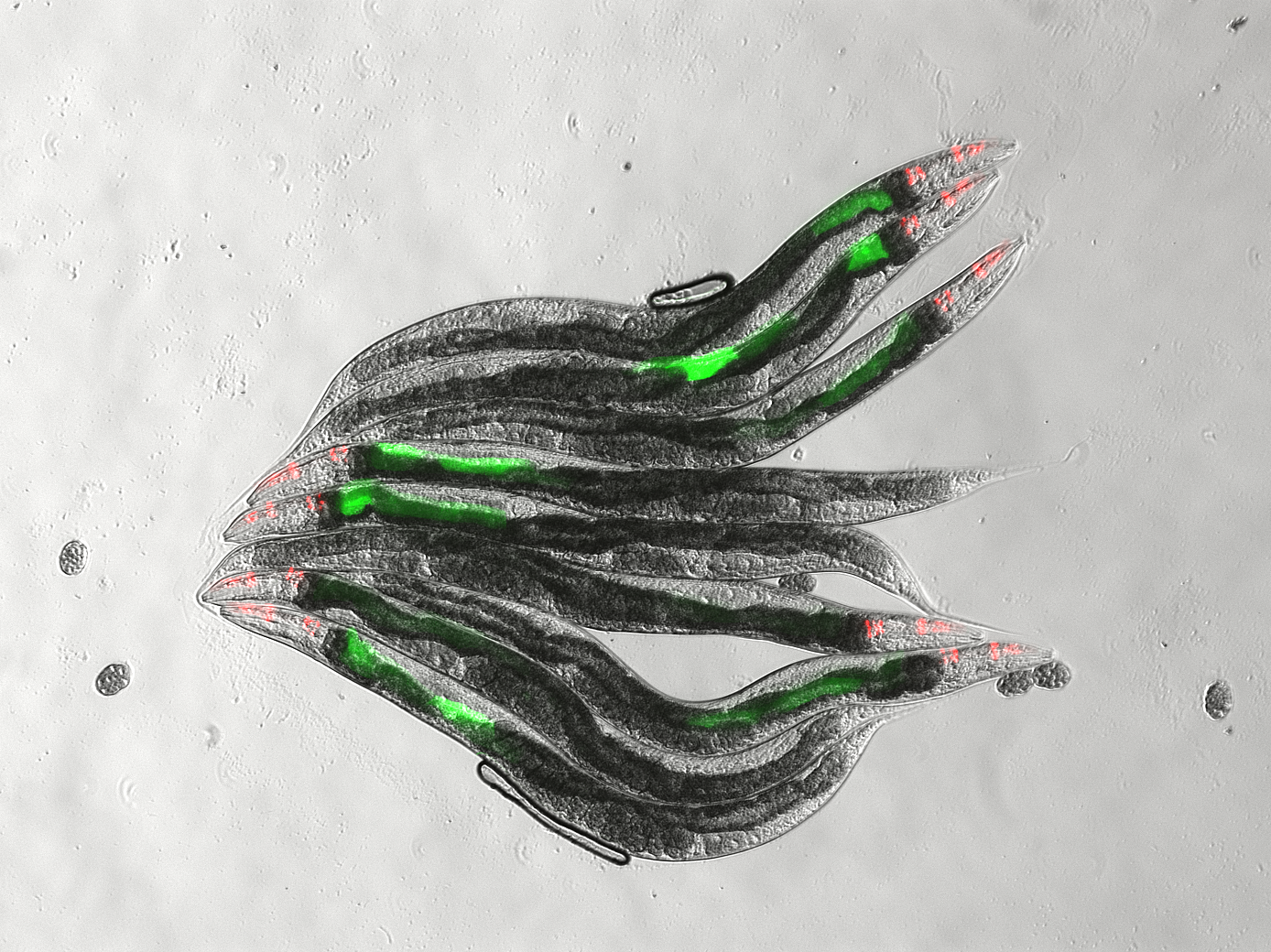

Supplement: Supplementary file 5 — Source data Fig. 2 [file 44319_2025_589_MOESM5_ESM.zip › EMBOR-2024-60913V3_Source-Data For Figure 2/2A/wildtype Orsay.tif]

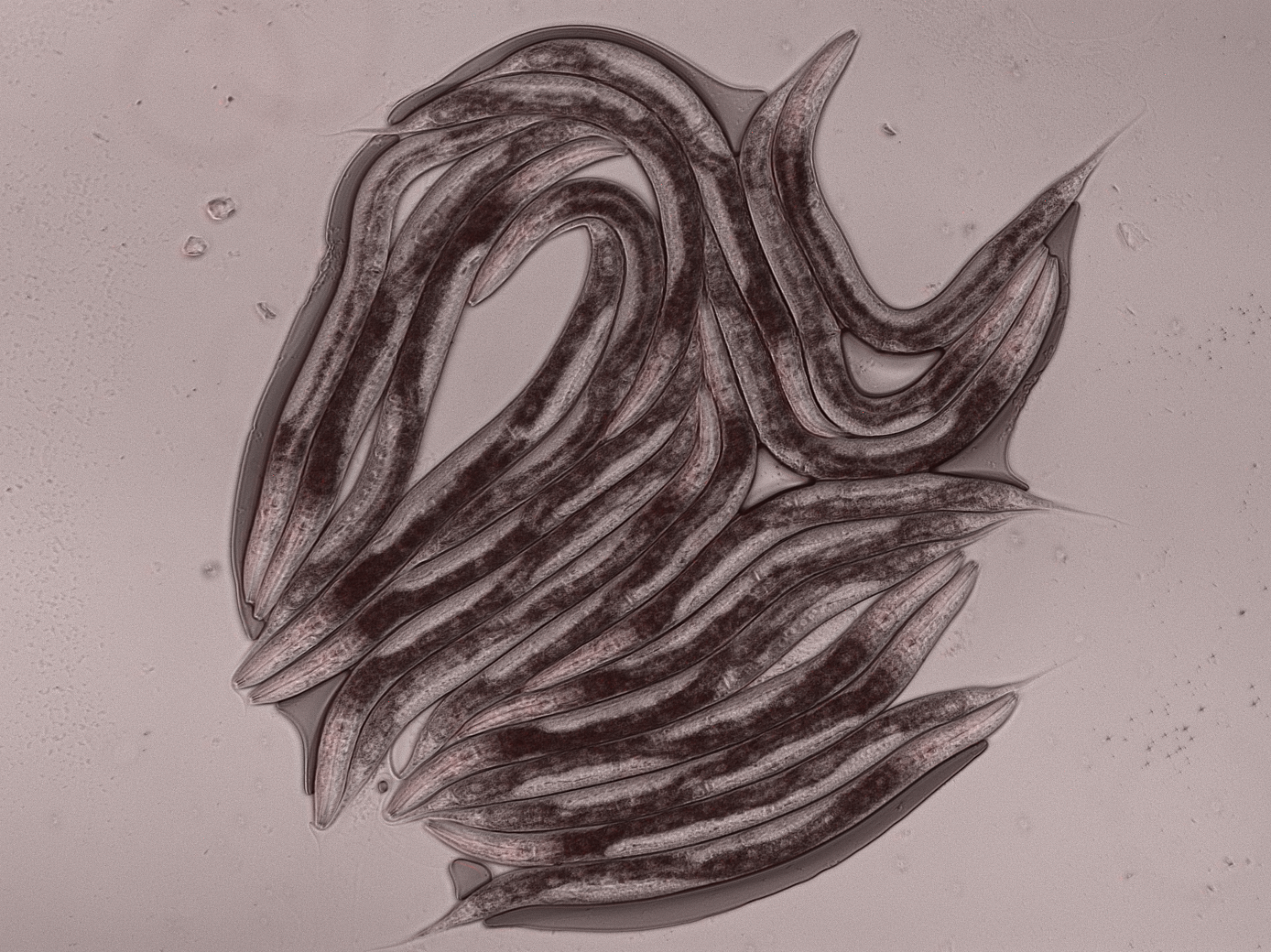

Supplement: Supplementary file 6 — Source data Fig. 3 [file 44319_2025_589_MOESM6_ESM.zip › EMBOR-2024-60913V2_Source-Data For Figure 3/3C/drh-1 RNAi.tif]

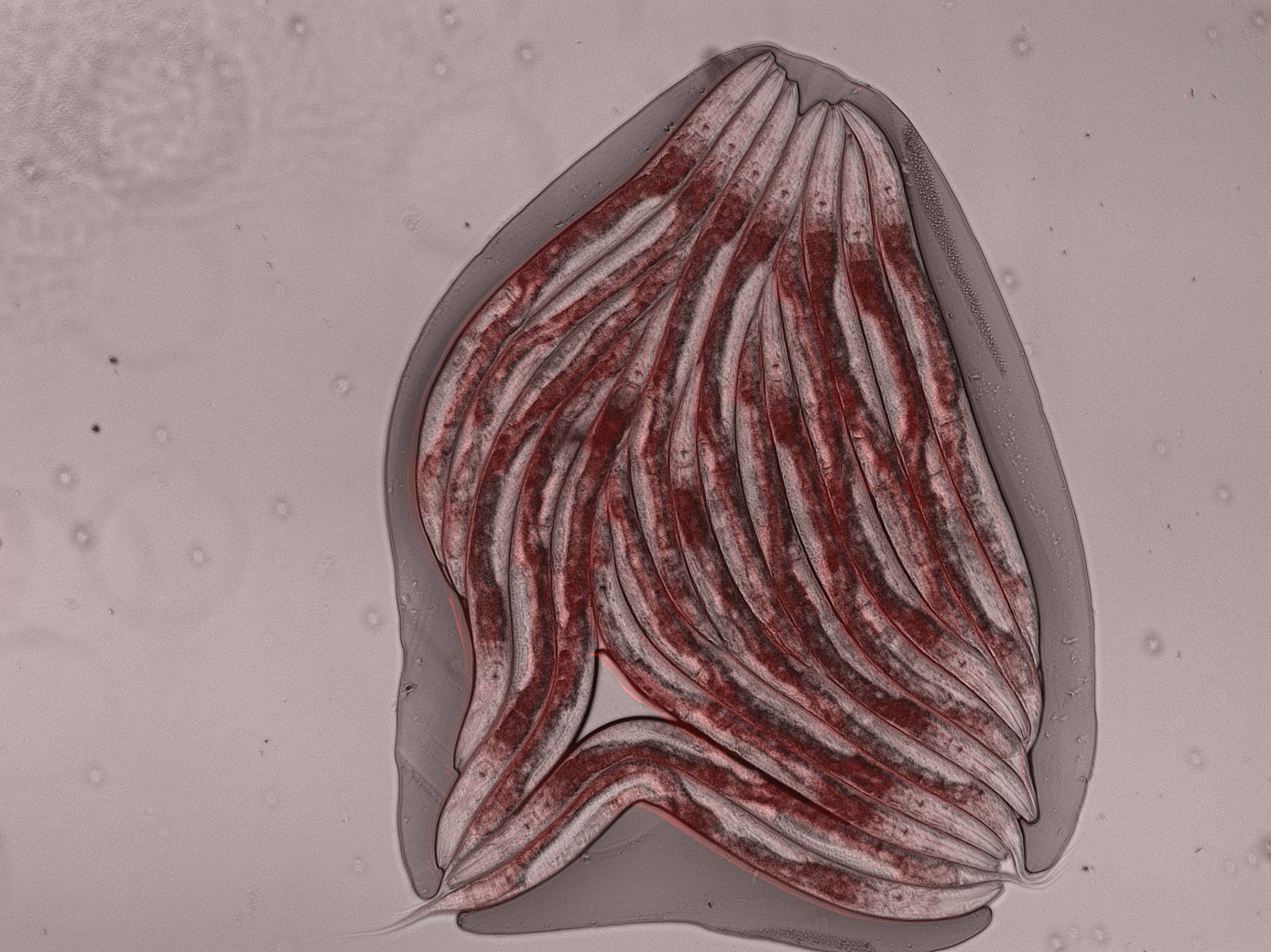

Supplement: Supplementary file 6 — Source data Fig. 3 [file 44319_2025_589_MOESM6_ESM.zip › EMBOR-2024-60913V2_Source-Data For Figure 3/3C/ev.tif]

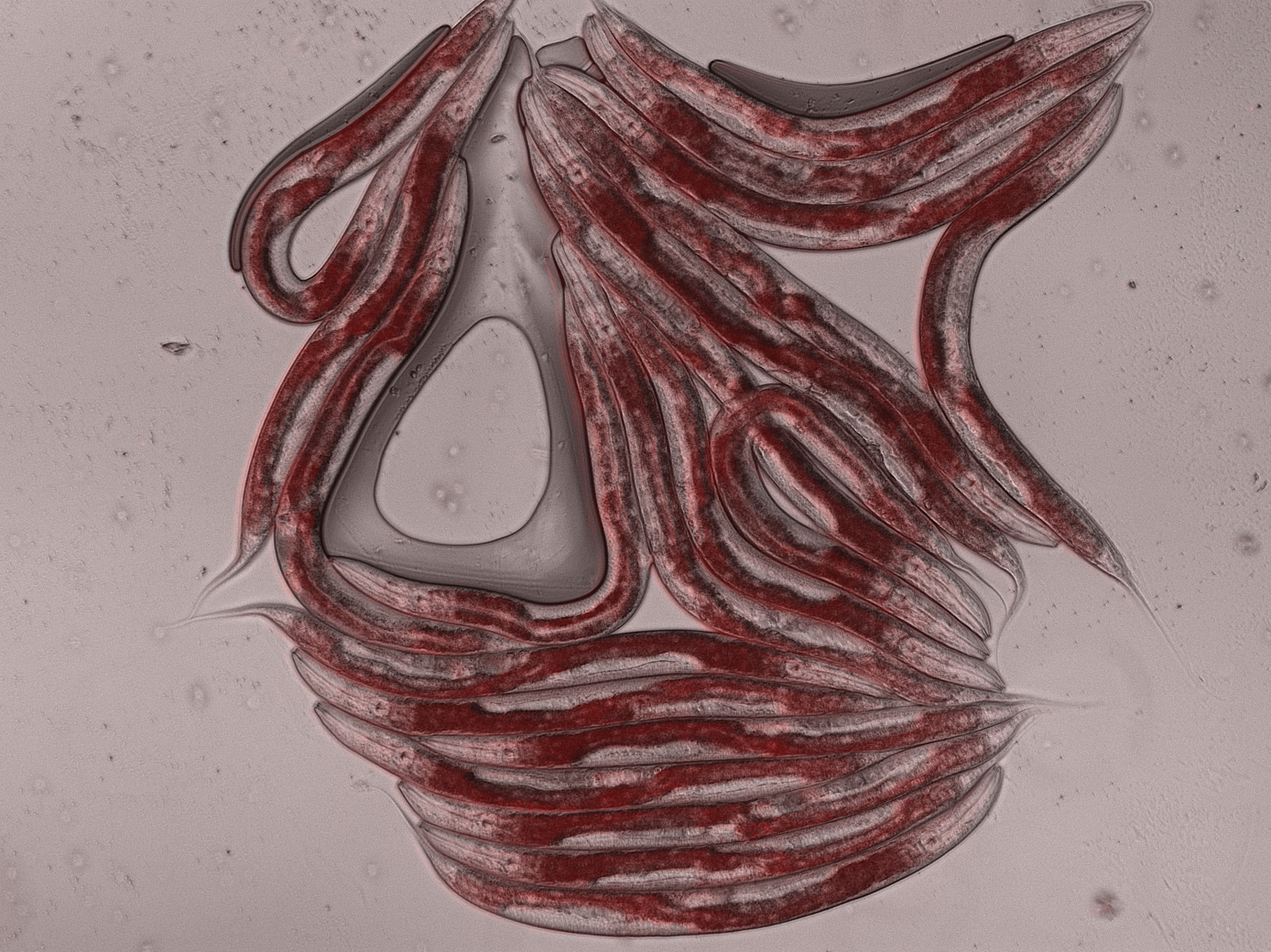

Supplement: Supplementary file 6 — Source data Fig. 3 [file 44319_2025_589_MOESM6_ESM.zip › EMBOR-2024-60913V2_Source-Data For Figure 3/3C/smo-1 RNAi.tif]

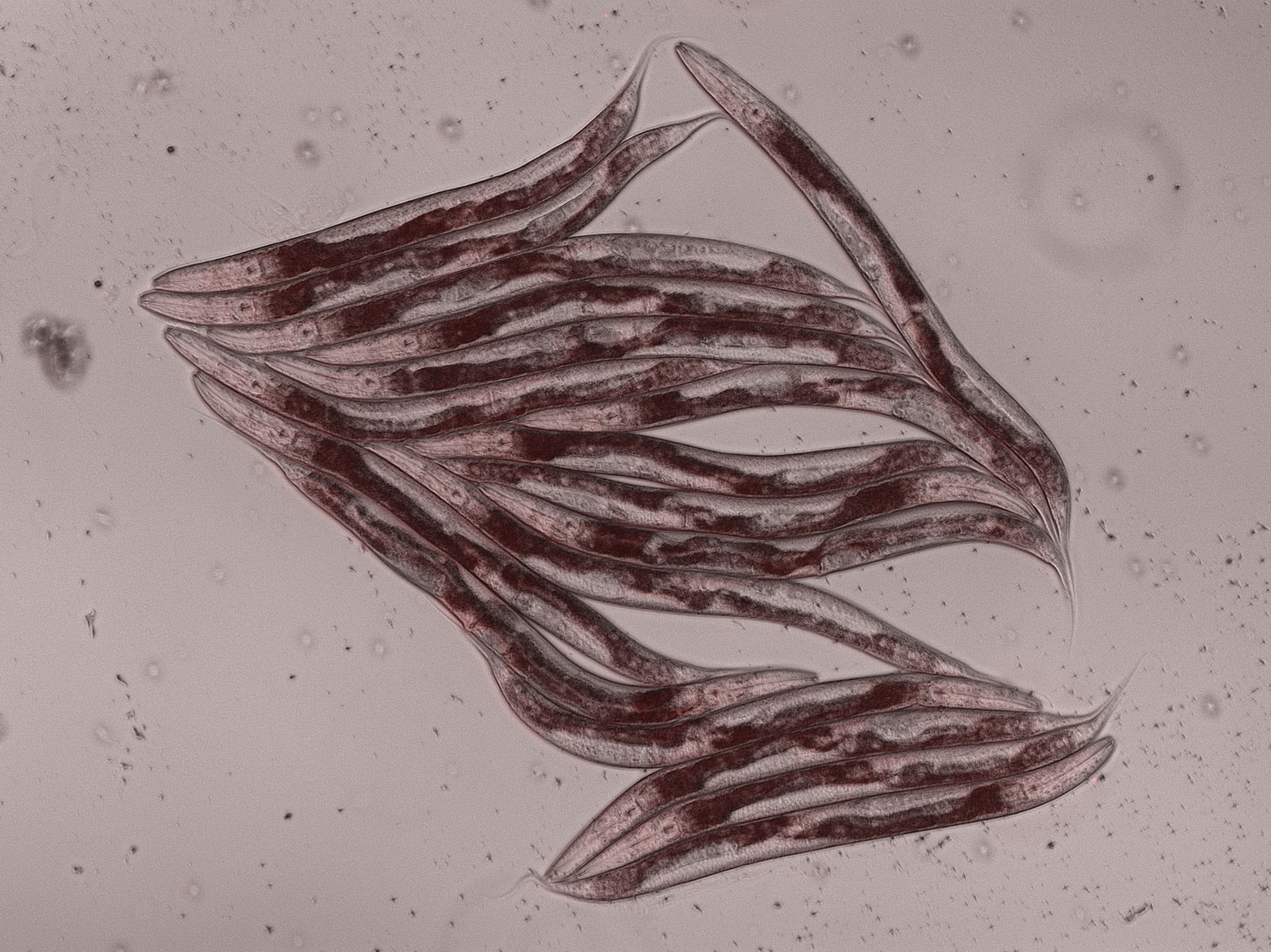

Supplement: Supplementary file 6 — Source data Fig. 3 [file 44319_2025_589_MOESM6_ESM.zip › EMBOR-2024-60913V2_Source-Data For Figure 3/3C/ulp-4 RNAi.tif]

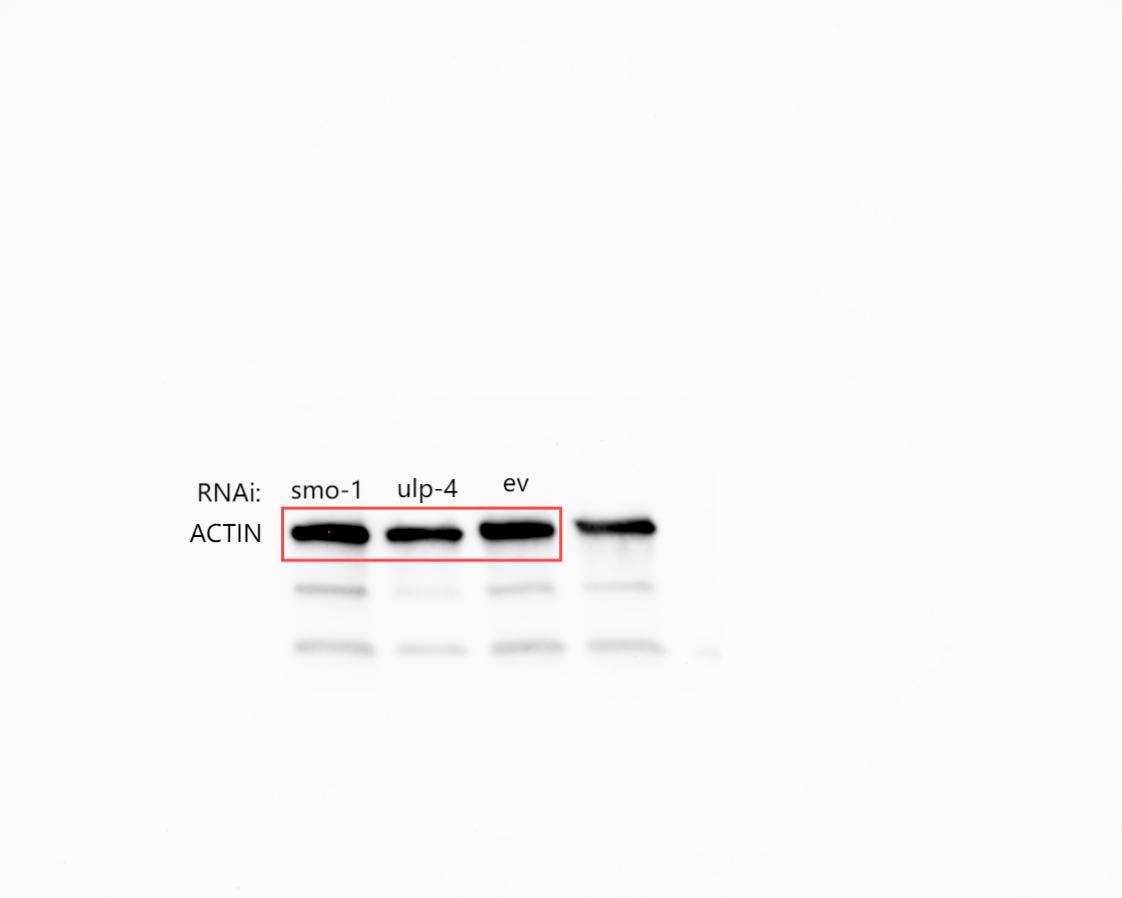

Supplement: Supplementary file 6 — Source data Fig. 3 [file 44319_2025_589_MOESM6_ESM.zip › EMBOR-2024-60913V2_Source-Data For Figure 3/3D/ACTIN.jpg]

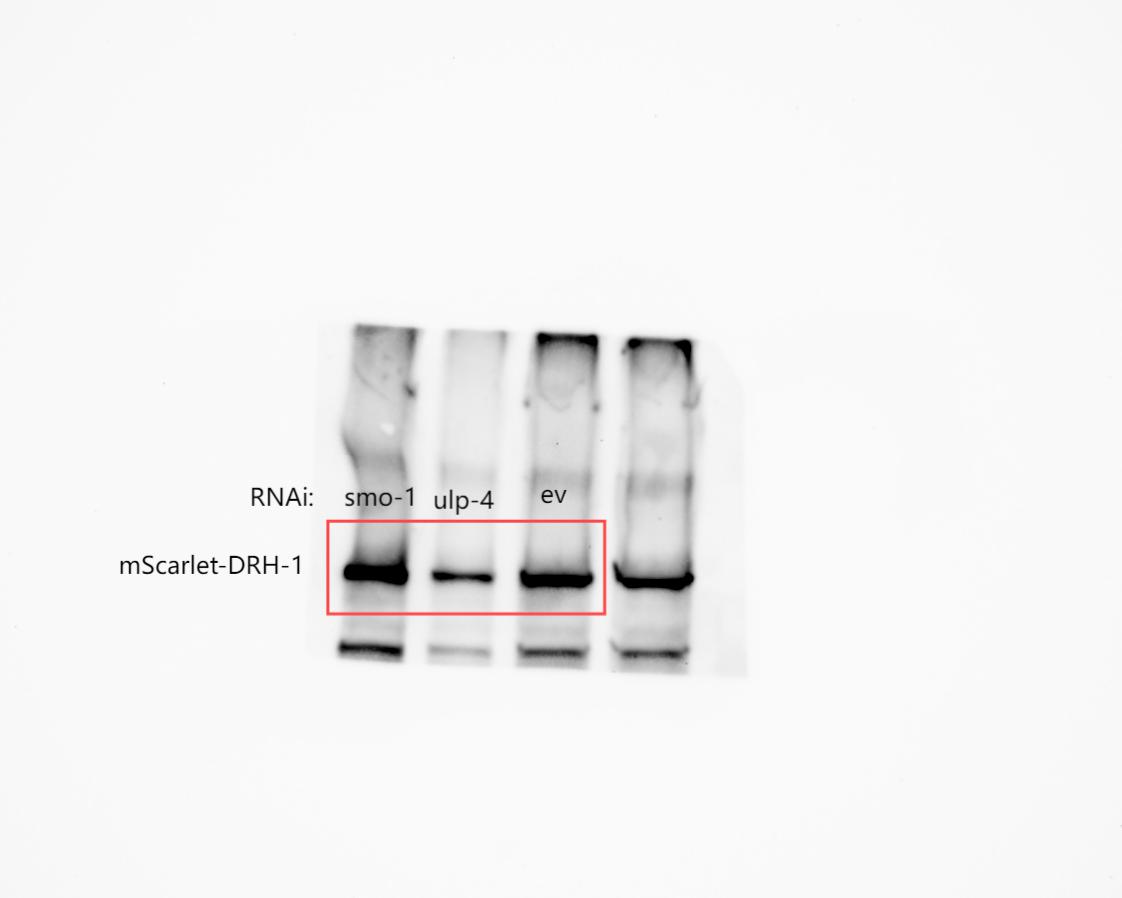

Supplement: Supplementary file 6 — Source data Fig. 3 [file 44319_2025_589_MOESM6_ESM.zip › EMBOR-2024-60913V2_Source-Data For Figure 3/3D/mScarlet-DRH-1.jpg]

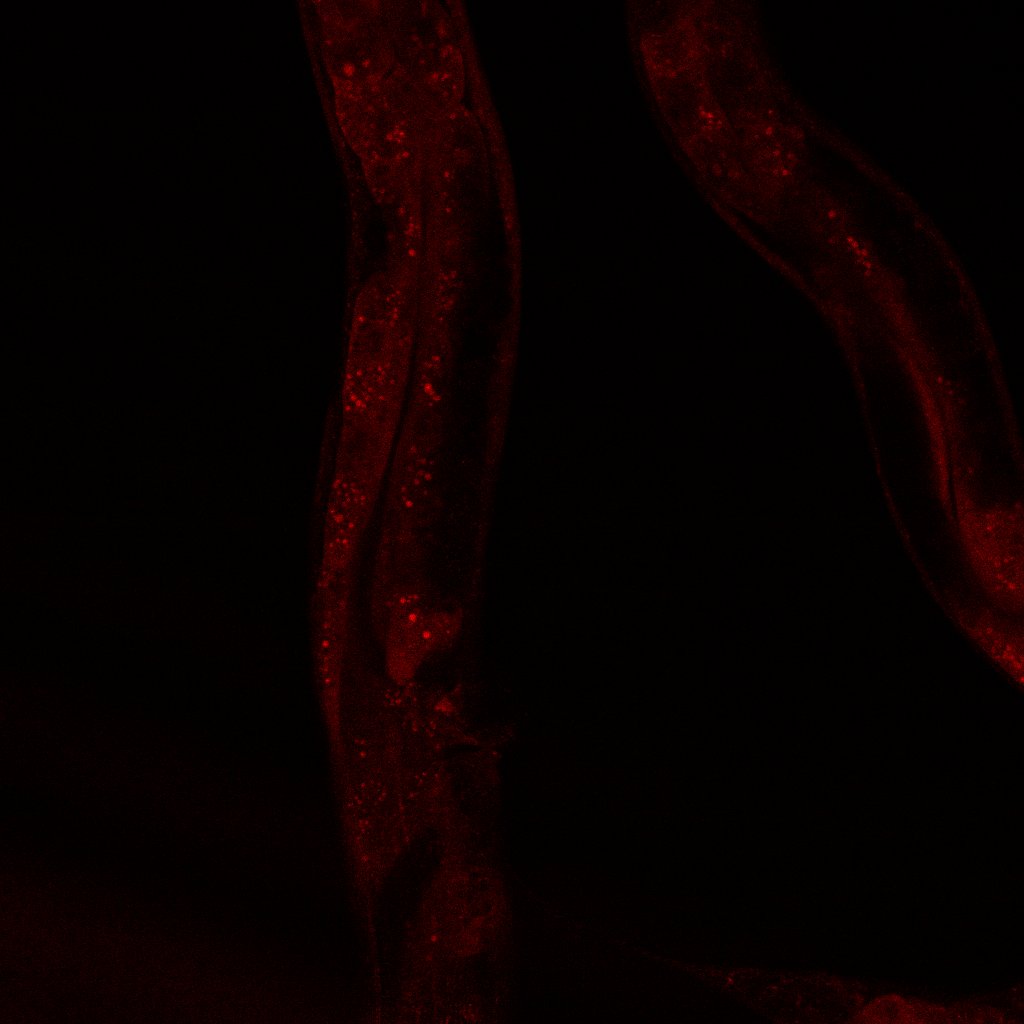

Supplement: Supplementary file 7 — Source data Fig. 4 [file 44319_2025_589_MOESM7_ESM.zip › EMBOR-2024-60913V2_Source-Data For Figure 4/4A/ulp-4(0) control.jpg]

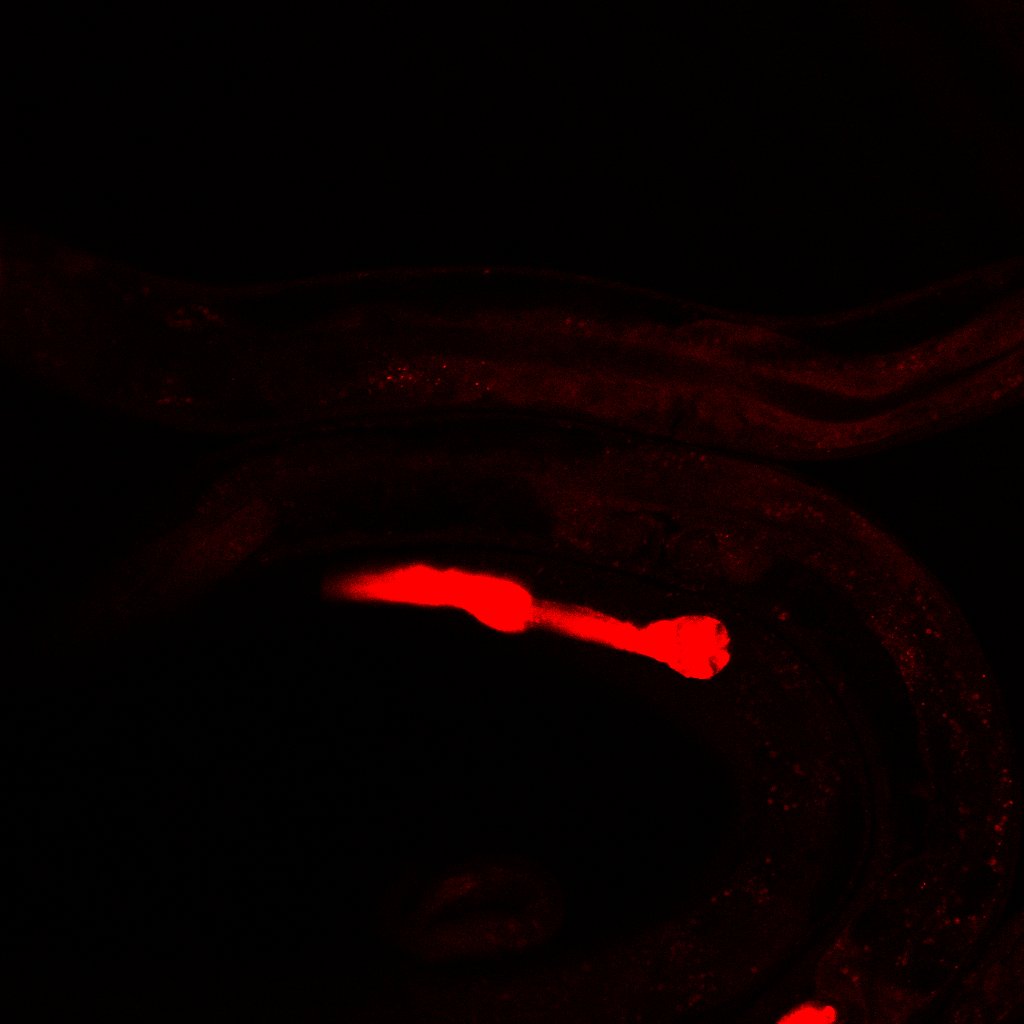

Supplement: Supplementary file 7 — Source data Fig. 4 [file 44319_2025_589_MOESM7_ESM.zip › EMBOR-2024-60913V2_Source-Data For Figure 4/4A/ulp-4(0) Orsay.jpg]

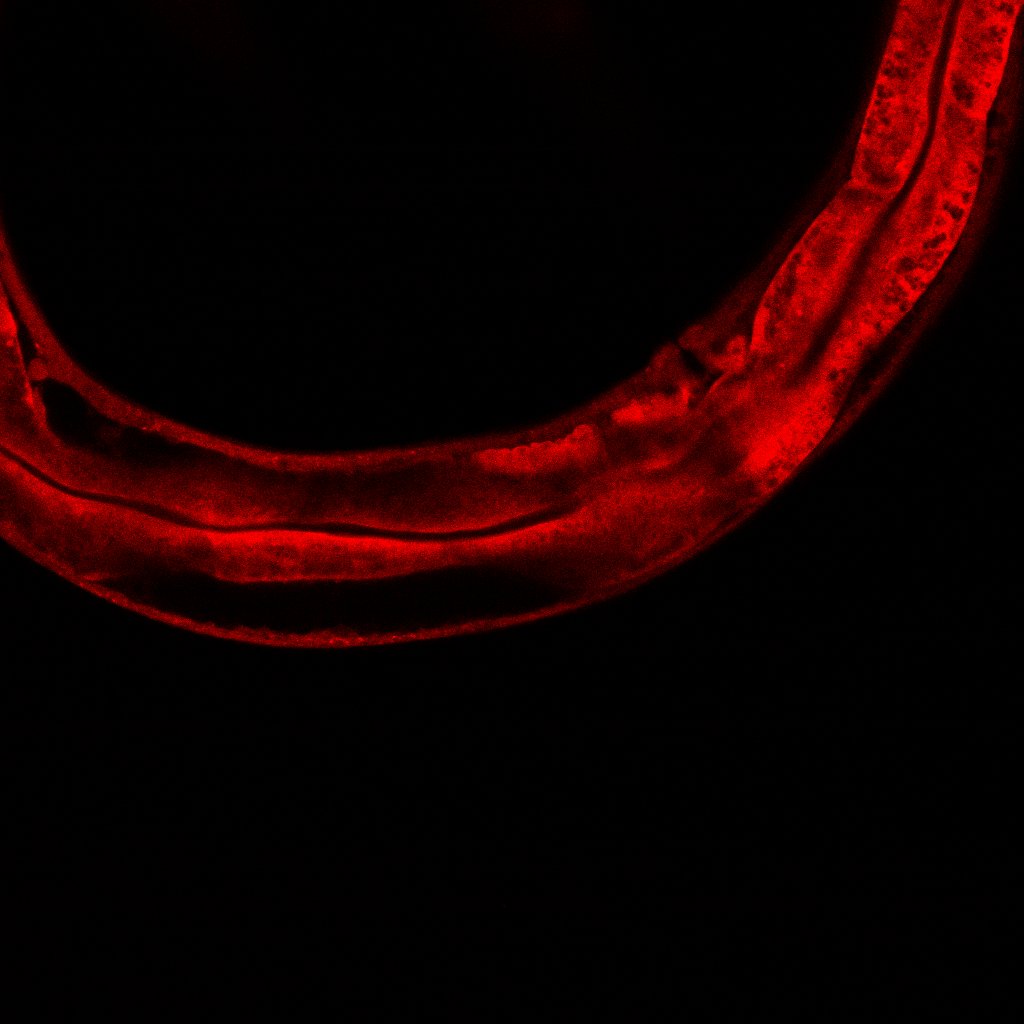

Supplement: Supplementary file 7 — Source data Fig. 4 [file 44319_2025_589_MOESM7_ESM.zip › EMBOR-2024-60913V2_Source-Data For Figure 4/4A/ulp-4(0); ulp-4 intestinal rescue control.jpg]

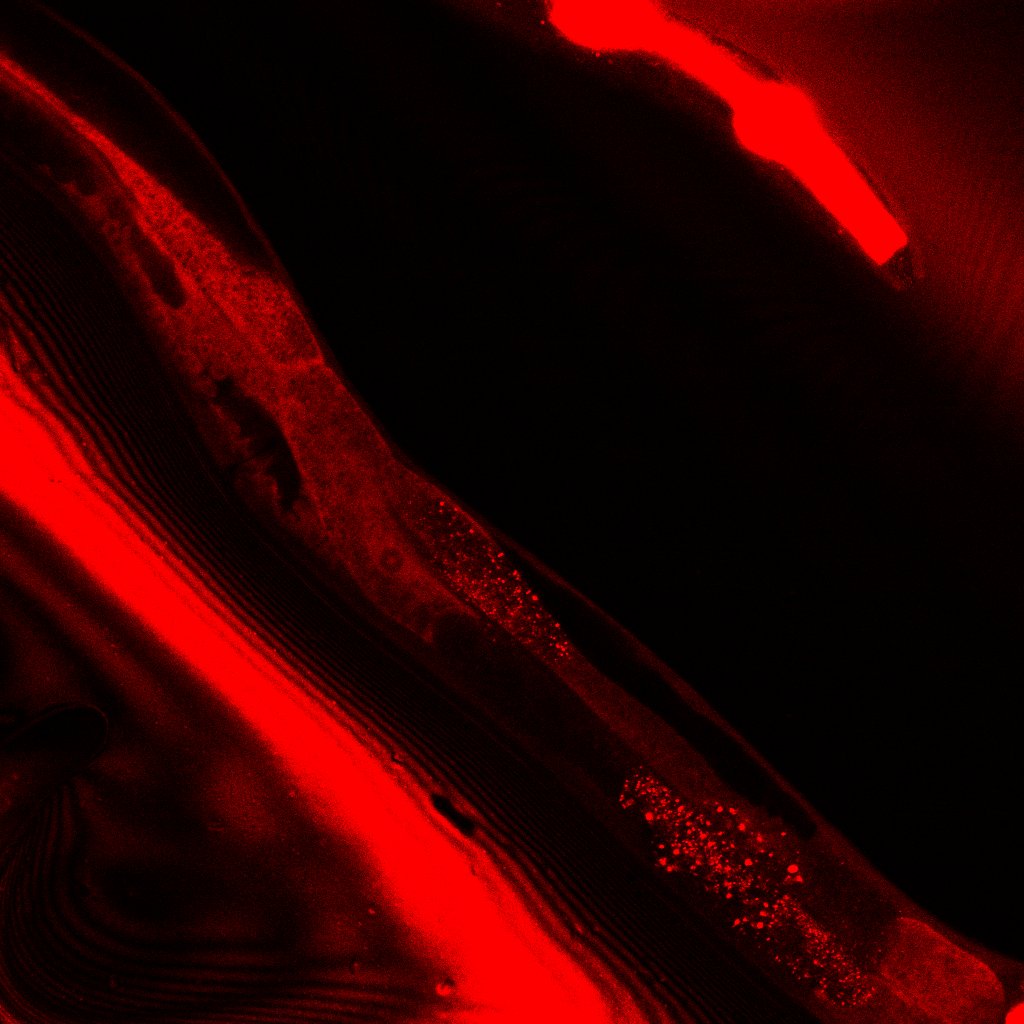

Supplement: Supplementary file 7 — Source data Fig. 4 [file 44319_2025_589_MOESM7_ESM.zip › EMBOR-2024-60913V2_Source-Data For Figure 4/4A/ulp-4(0); ulp-4 intestinal rescue Orsay.jpg]

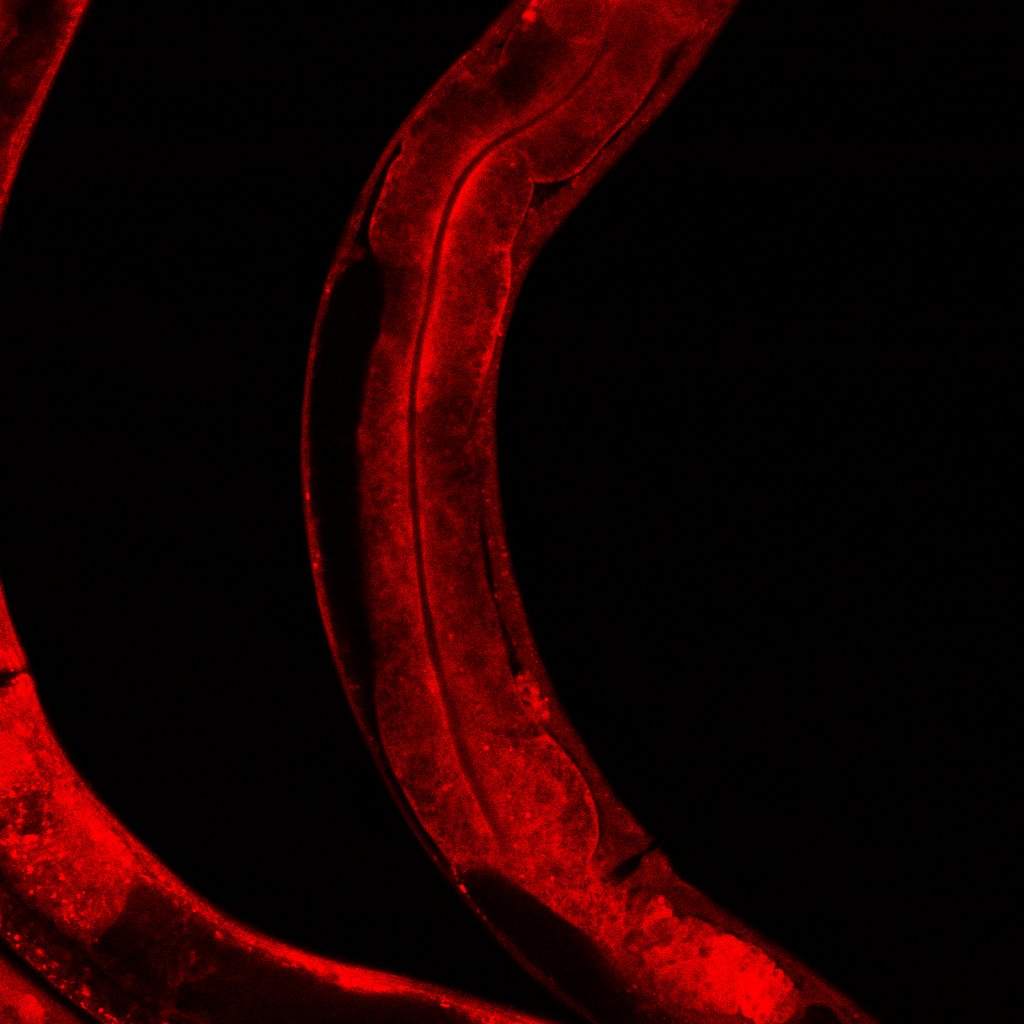

Supplement: Supplementary file 7 — Source data Fig. 4 [file 44319_2025_589_MOESM7_ESM.zip › EMBOR-2024-60913V2_Source-Data For Figure 4/4A/wildtype control.jpg]

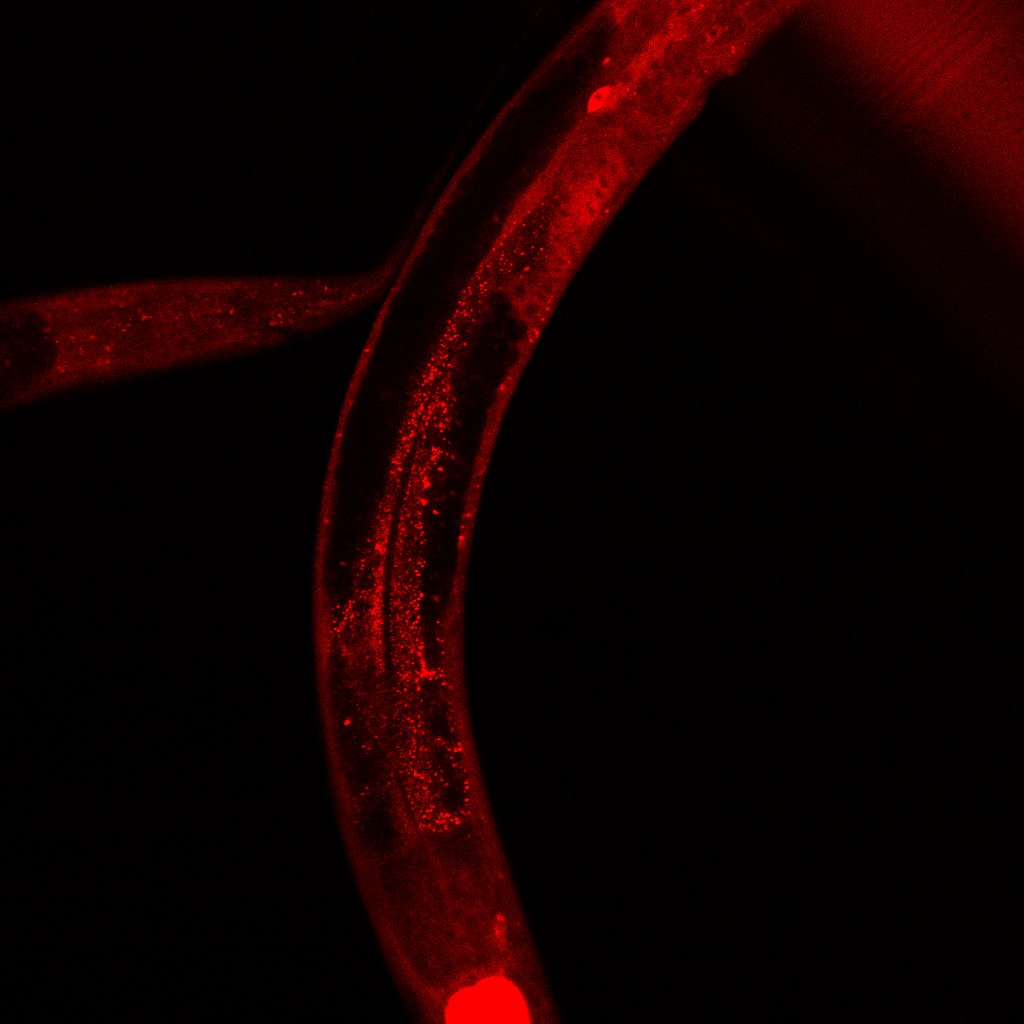

Supplement: Supplementary file 7 — Source data Fig. 4 [file 44319_2025_589_MOESM7_ESM.zip › EMBOR-2024-60913V2_Source-Data For Figure 4/4A/wildtype orsay.jpg]

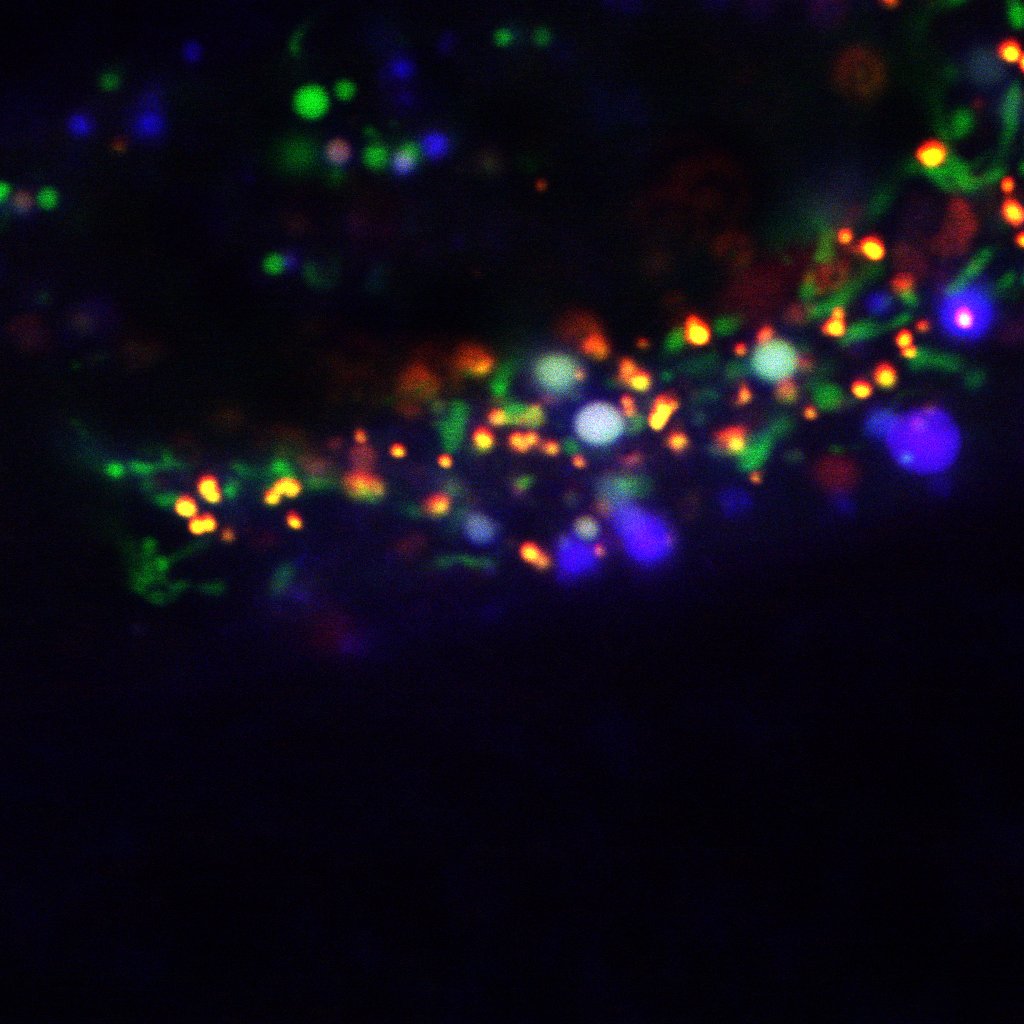

Supplement: Supplementary file 7 — Source data Fig. 4 [file 44319_2025_589_MOESM7_ESM.zip › EMBOR-2024-60913V2_Source-Data For Figure 4/4B/mito-GFP; mScarlet-DRH-1 infected with Orsay.jpg]

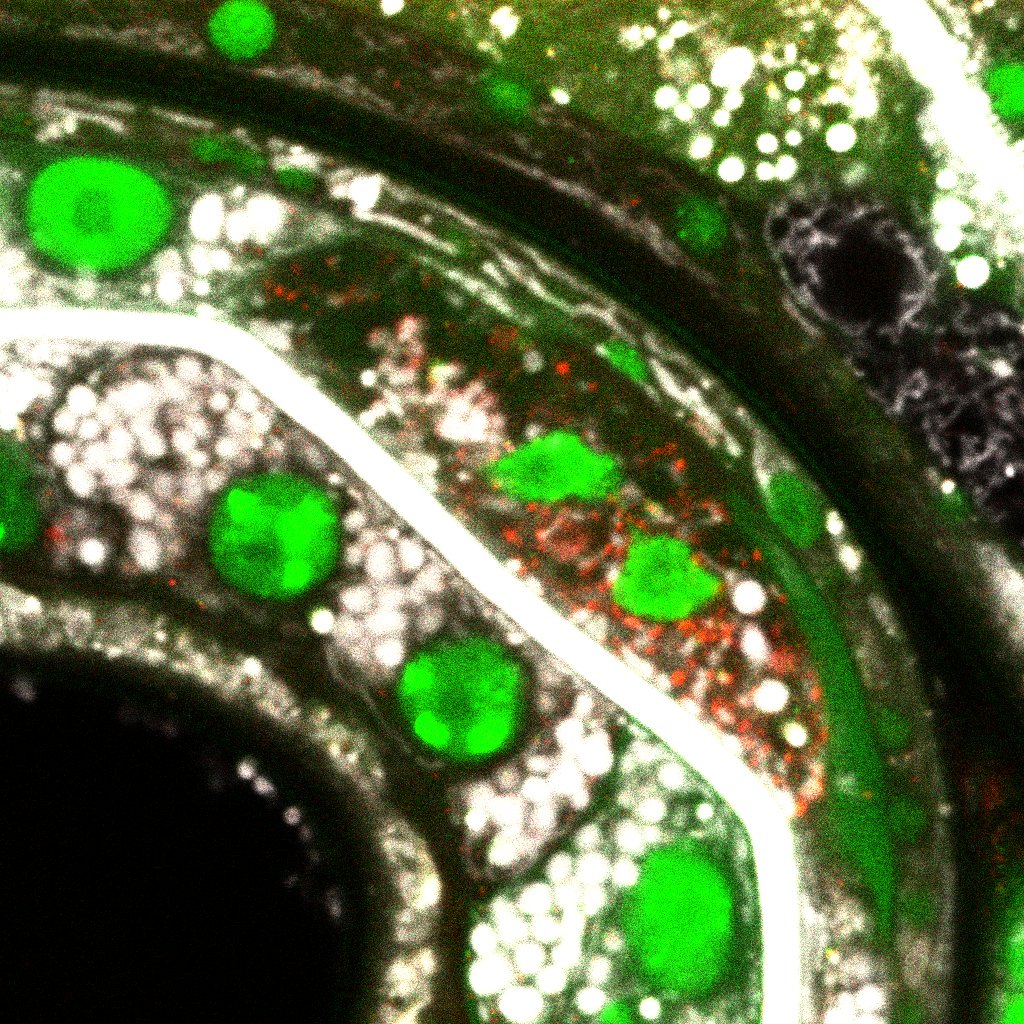

Supplement: Supplementary file 7 — Source data Fig. 4 [file 44319_2025_589_MOESM7_ESM.zip › EMBOR-2024-60913V2_Source-Data For Figure 4/4C/GFP-SMO-1; mScarlet-DRH-1 infected with Orsay, staining with far red mitotracker.jpg]

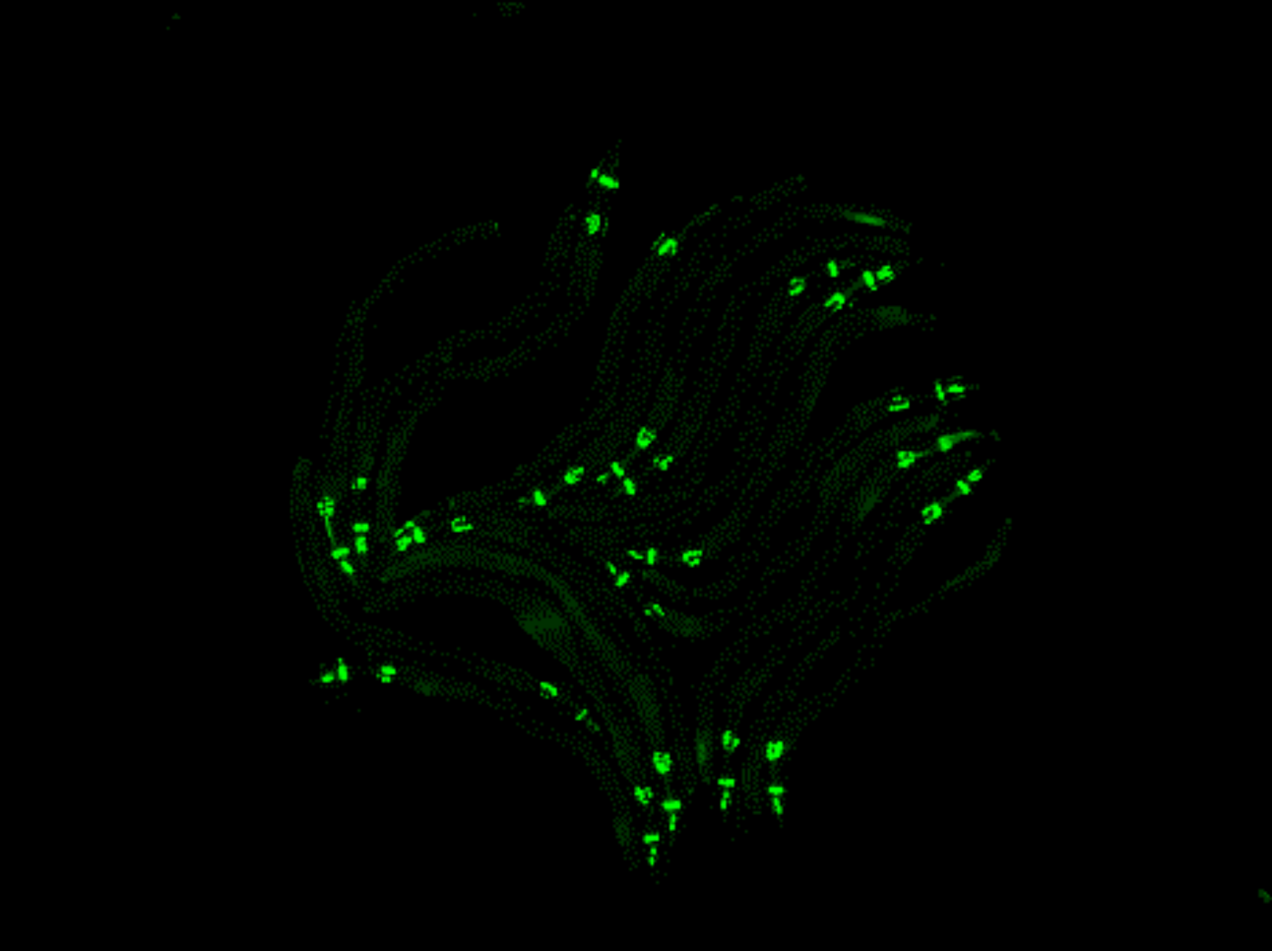

Supplement: Supplementary file 8 — Source data Fig. 5 [file 44319_2025_589_MOESM8_ESM.zip › EMBOR-2024-60913V3_Source-Data For Figure 5/5A/mScarlet-DRH-1 OE ulp-4 RNAi pals-5-gfp induction.tif]

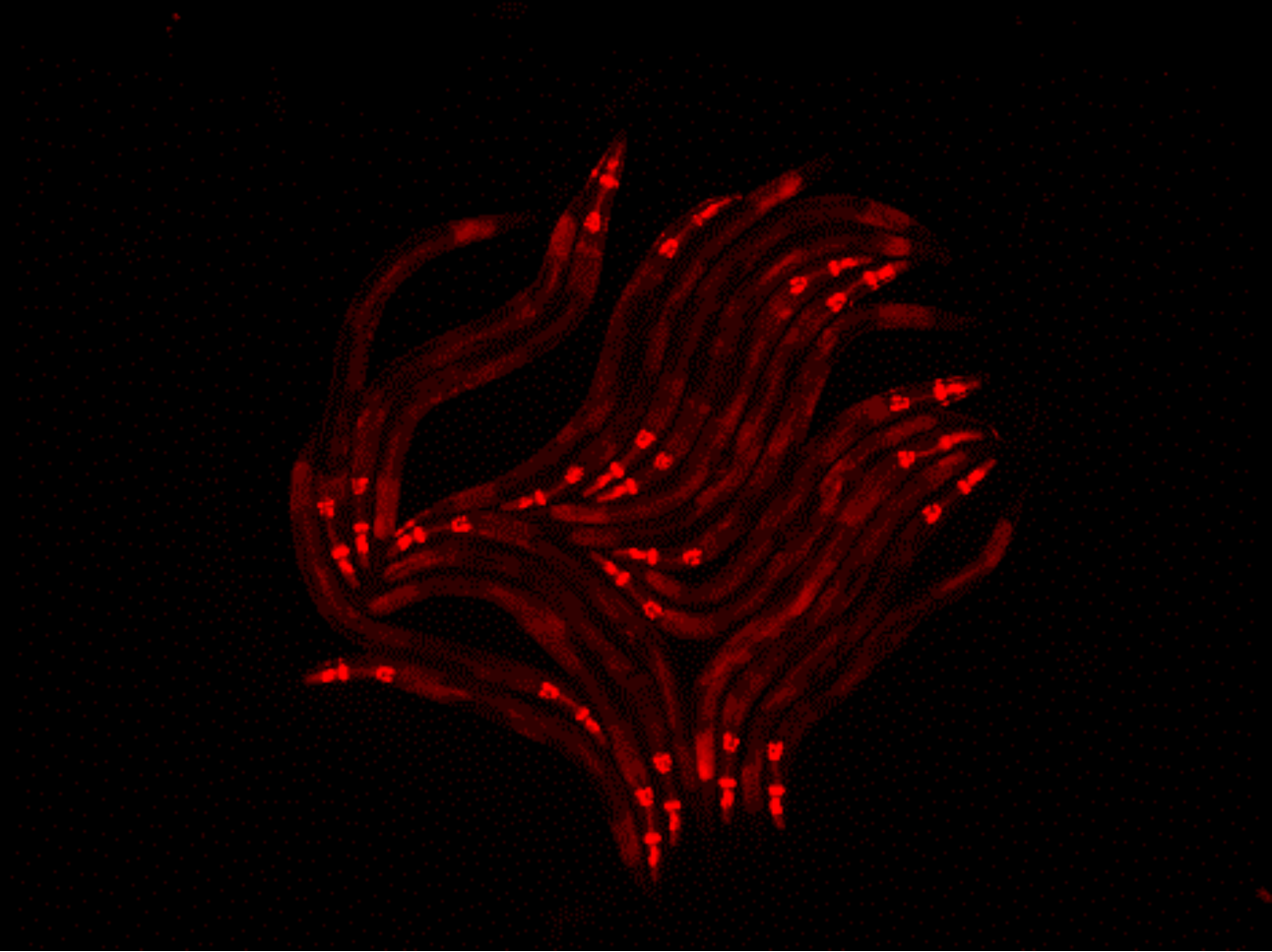

Supplement: Supplementary file 8 — Source data Fig. 5 [file 44319_2025_589_MOESM8_ESM.zip › EMBOR-2024-60913V3_Source-Data For Figure 5/5A/mScarlet-DRH-1 OE ulp-4 RNAi.tif]

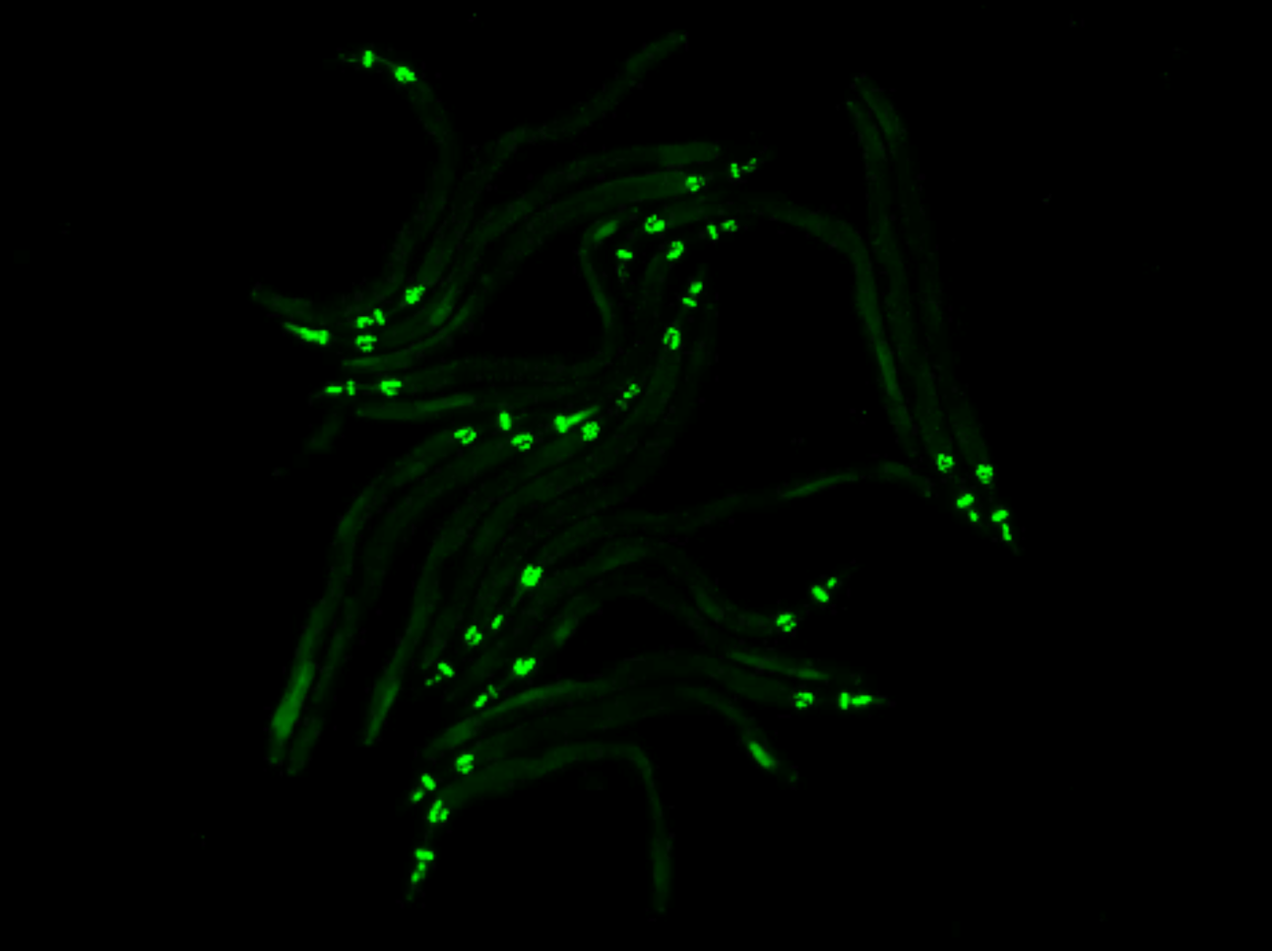

Supplement: Supplementary file 8 — Source data Fig. 5 [file 44319_2025_589_MOESM8_ESM.zip › EMBOR-2024-60913V3_Source-Data For Figure 5/5A/mScarlet-DRH-1OE ev pals-5-GFP.tif]

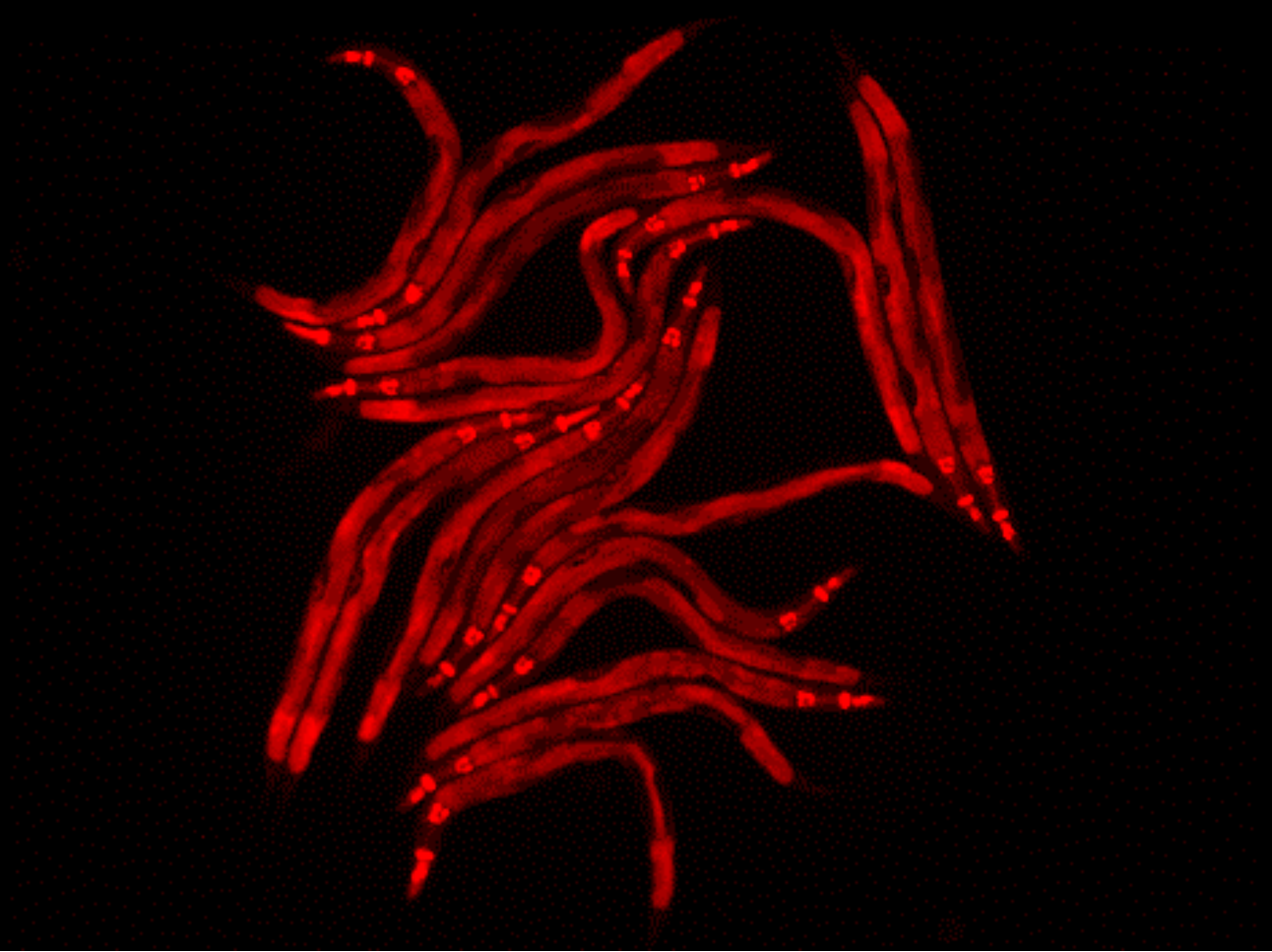

Supplement: Supplementary file 8 — Source data Fig. 5 [file 44319_2025_589_MOESM8_ESM.zip › EMBOR-2024-60913V3_Source-Data For Figure 5/5A/mScarlet-DRH-1OE ev.tif]

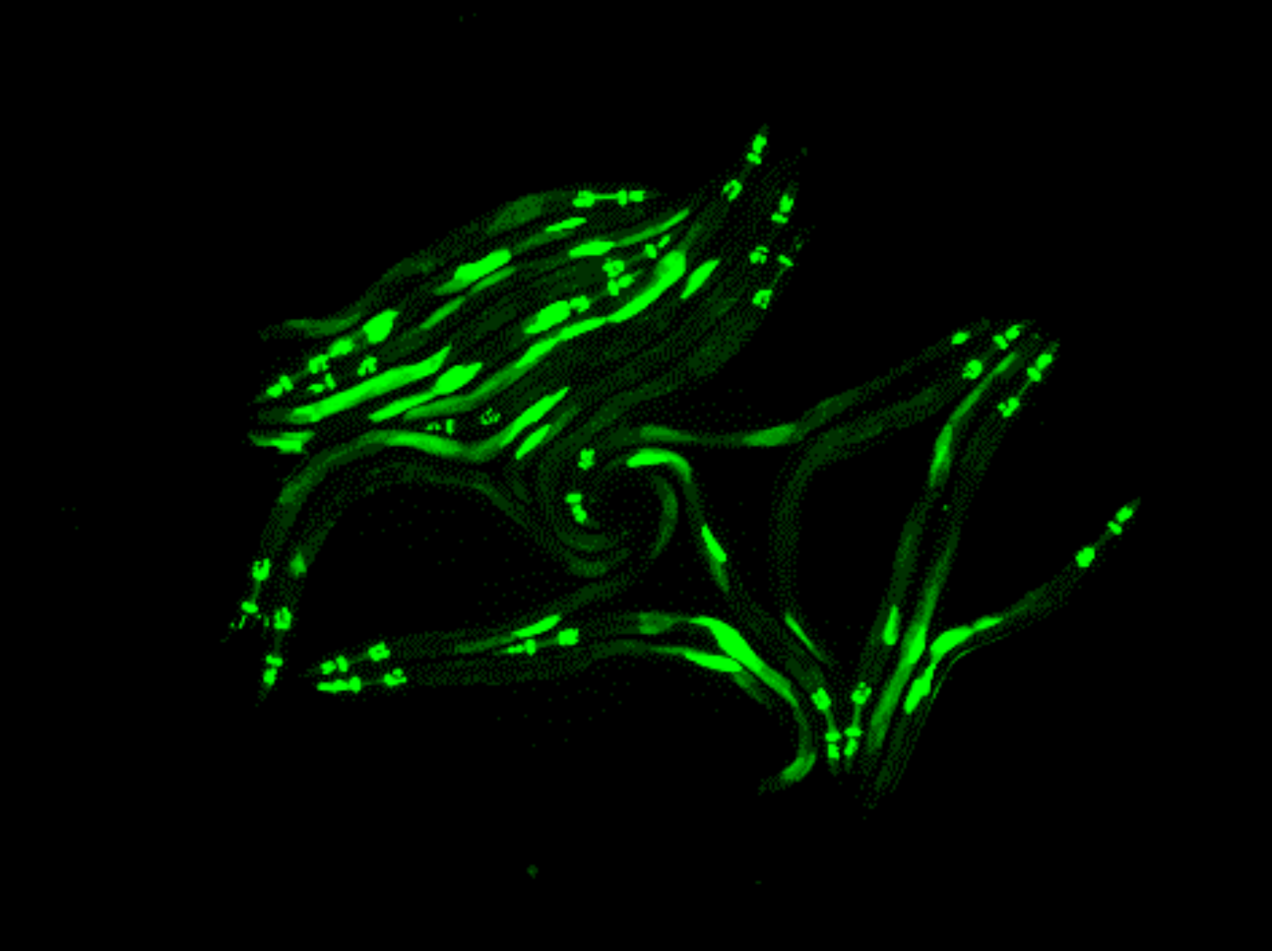

Supplement: Supplementary file 8 — Source data Fig. 5 [file 44319_2025_589_MOESM8_ESM.zip › EMBOR-2024-60913V3_Source-Data For Figure 5/5B/mScarlet-DRH-1(2KR) OE ev pals-5-gfp induction.tif]

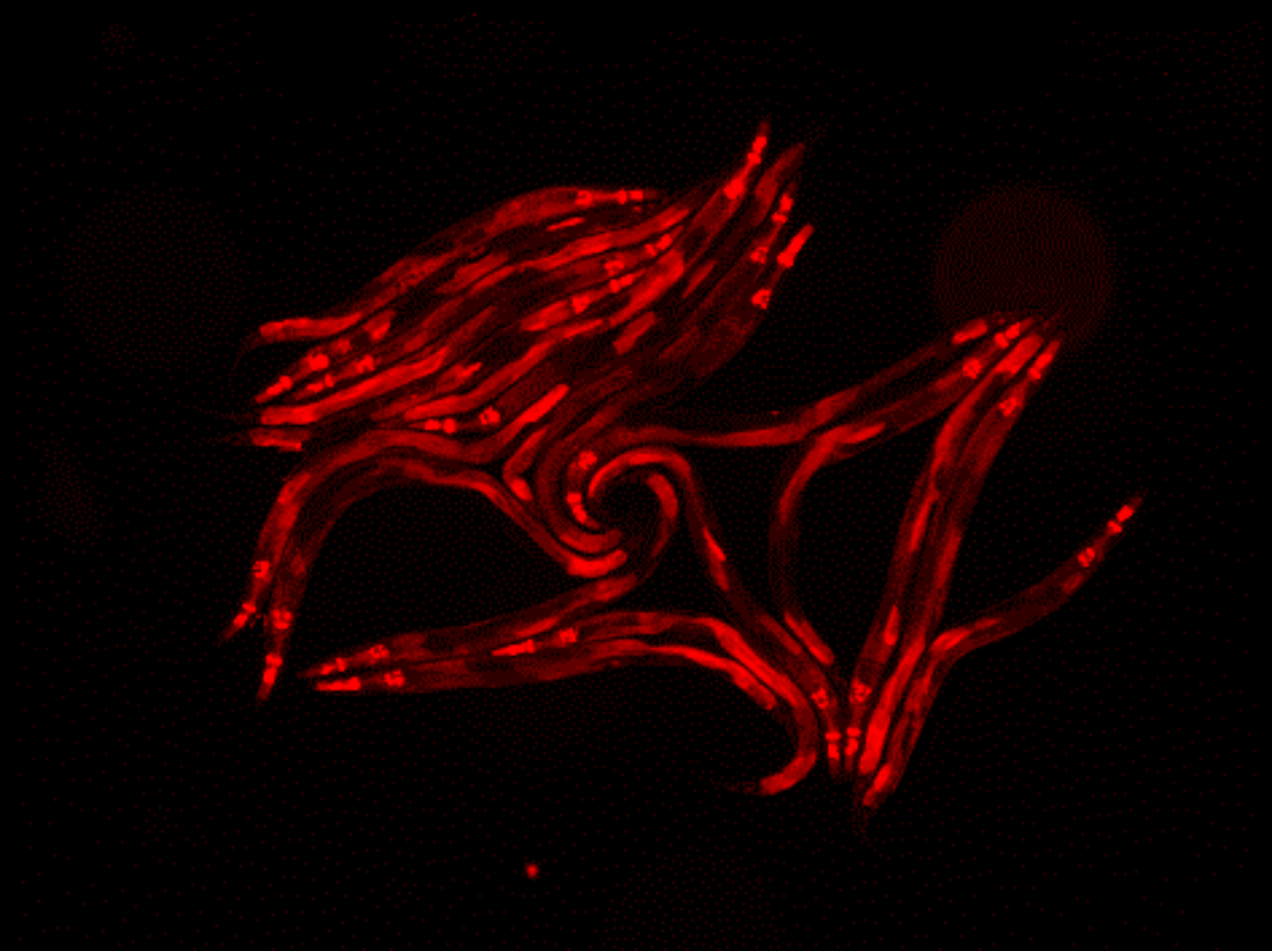

Supplement: Supplementary file 8 — Source data Fig. 5 [file 44319_2025_589_MOESM8_ESM.zip › EMBOR-2024-60913V3_Source-Data For Figure 5/5B/mScarlet-DRH-1(2KR) OE ev.tif]

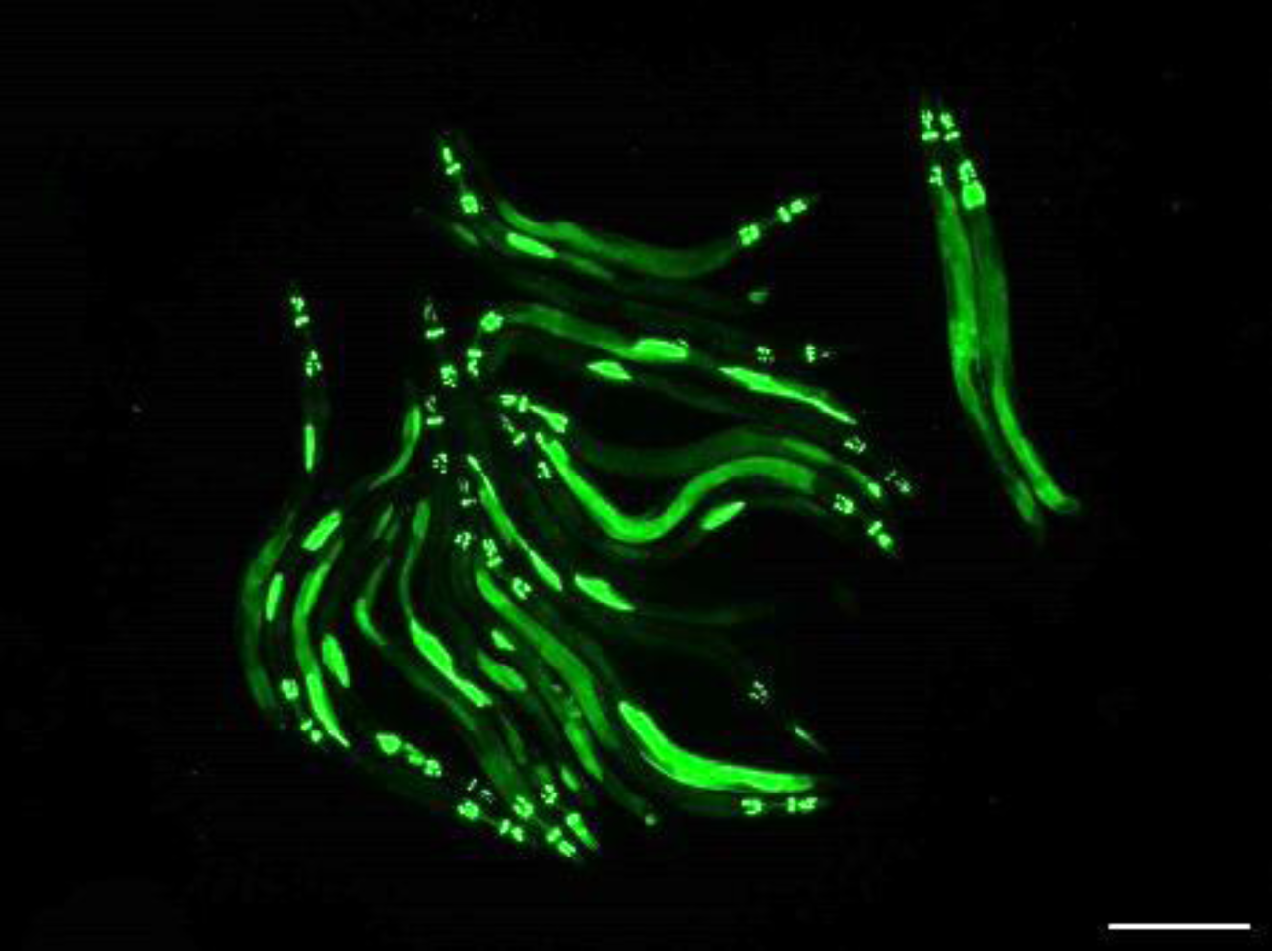

Supplement: Supplementary file 8 — Source data Fig. 5 [file 44319_2025_589_MOESM8_ESM.zip › EMBOR-2024-60913V3_Source-Data For Figure 5/5B/mScarlet-DRH-1(2KR) OE ulp-4 RNAi pals-5-gfp induction.tif]

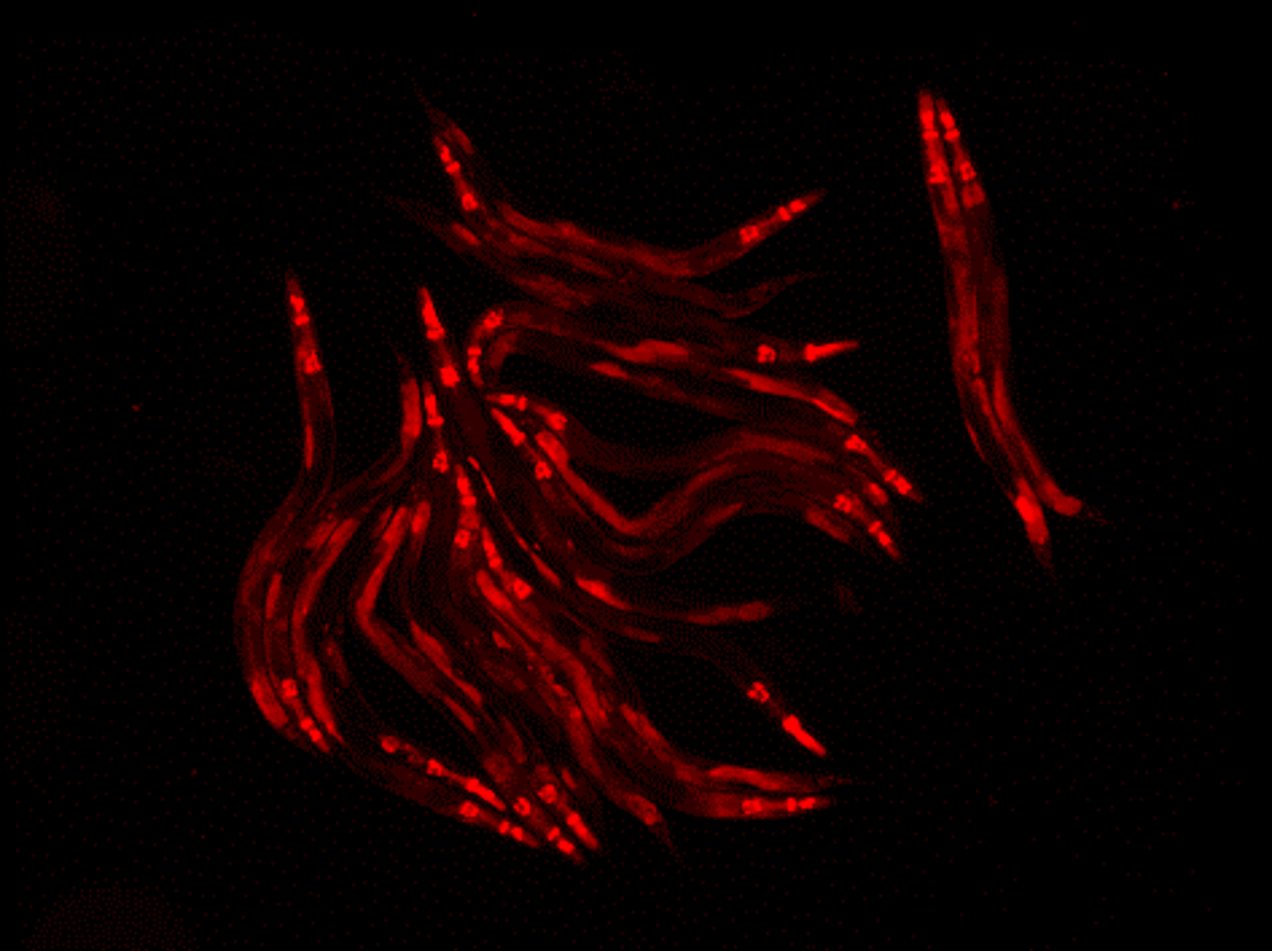

Supplement: Supplementary file 8 — Source data Fig. 5 [file 44319_2025_589_MOESM8_ESM.zip › EMBOR-2024-60913V3_Source-Data For Figure 5/5B/mScarlet-DRH-1(2KR) OE ulp-4 RNAi.tif]

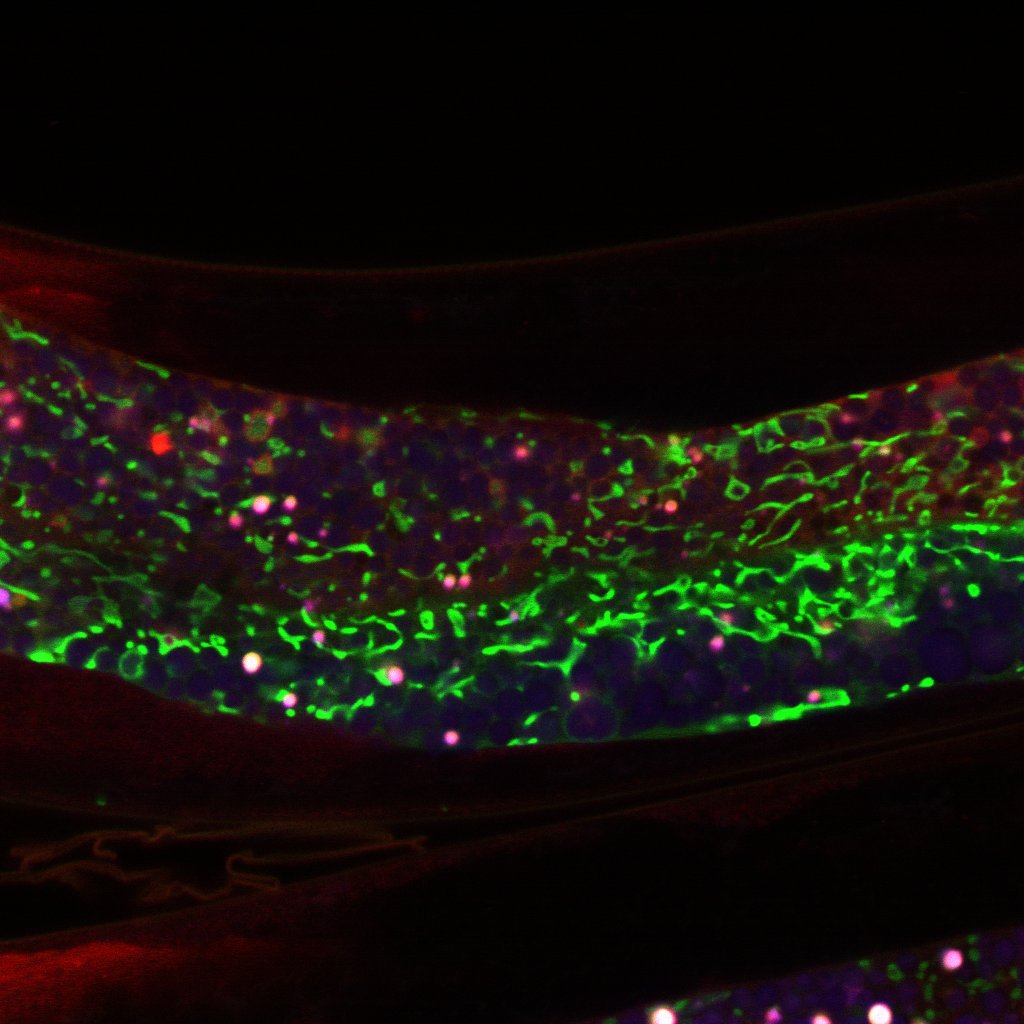

Supplement: Supplementary file 8 — Source data Fig. 5 [file 44319_2025_589_MOESM8_ESM.zip › EMBOR-2024-60913V3_Source-Data For Figure 5/5E/mScarlet-DRH-1(2KR);GFP-MITO.jpg]

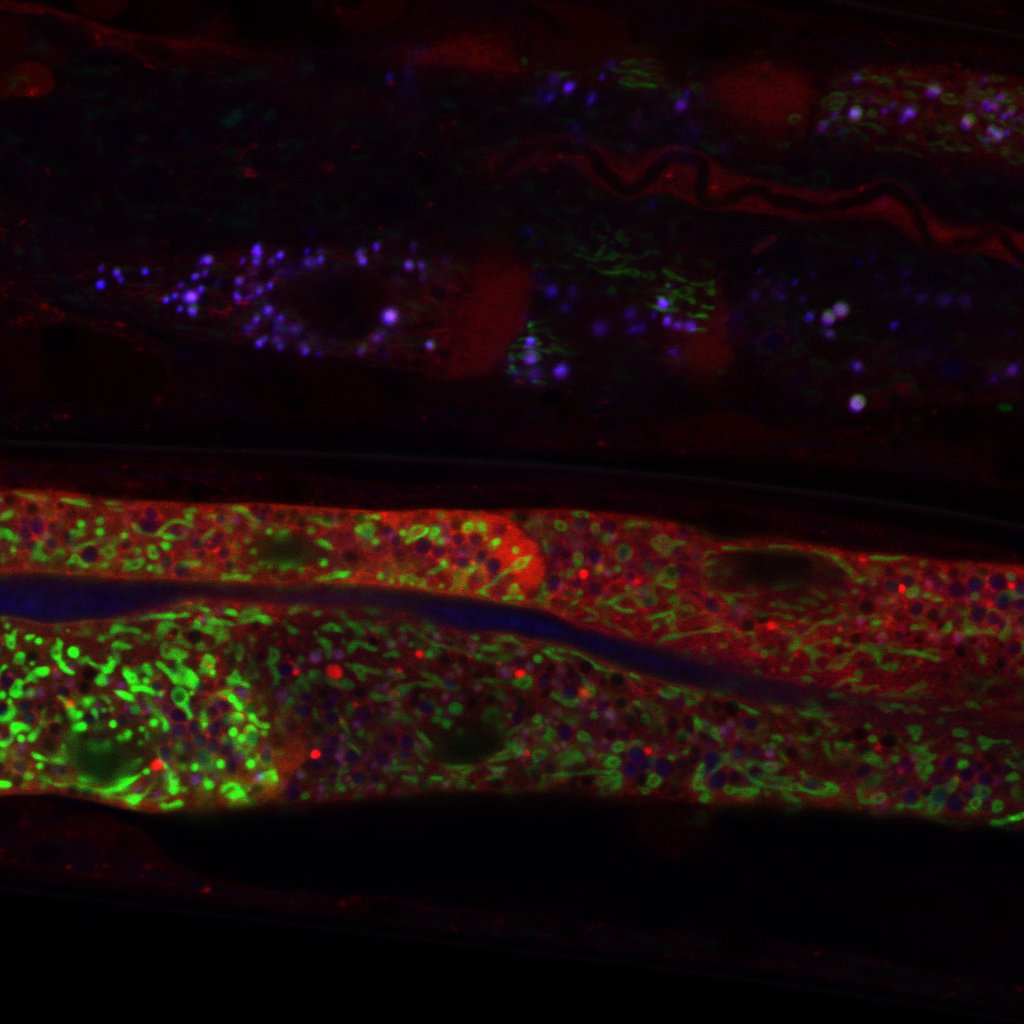

Supplement: Supplementary file 8 — Source data Fig. 5 [file 44319_2025_589_MOESM8_ESM.zip › EMBOR-2024-60913V3_Source-Data For Figure 5/5E/mScarlet-DRH-1;GFP-MITO.jpg]

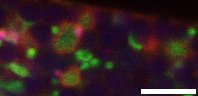

Supplement: Supplementary file 8 — Source data Fig. 5 [file 44319_2025_589_MOESM8_ESM.zip › EMBOR-2024-60913V3_Source-Data For Figure 5/5G/DRH-1 2KR enlarge 5um scale bar.jpg]

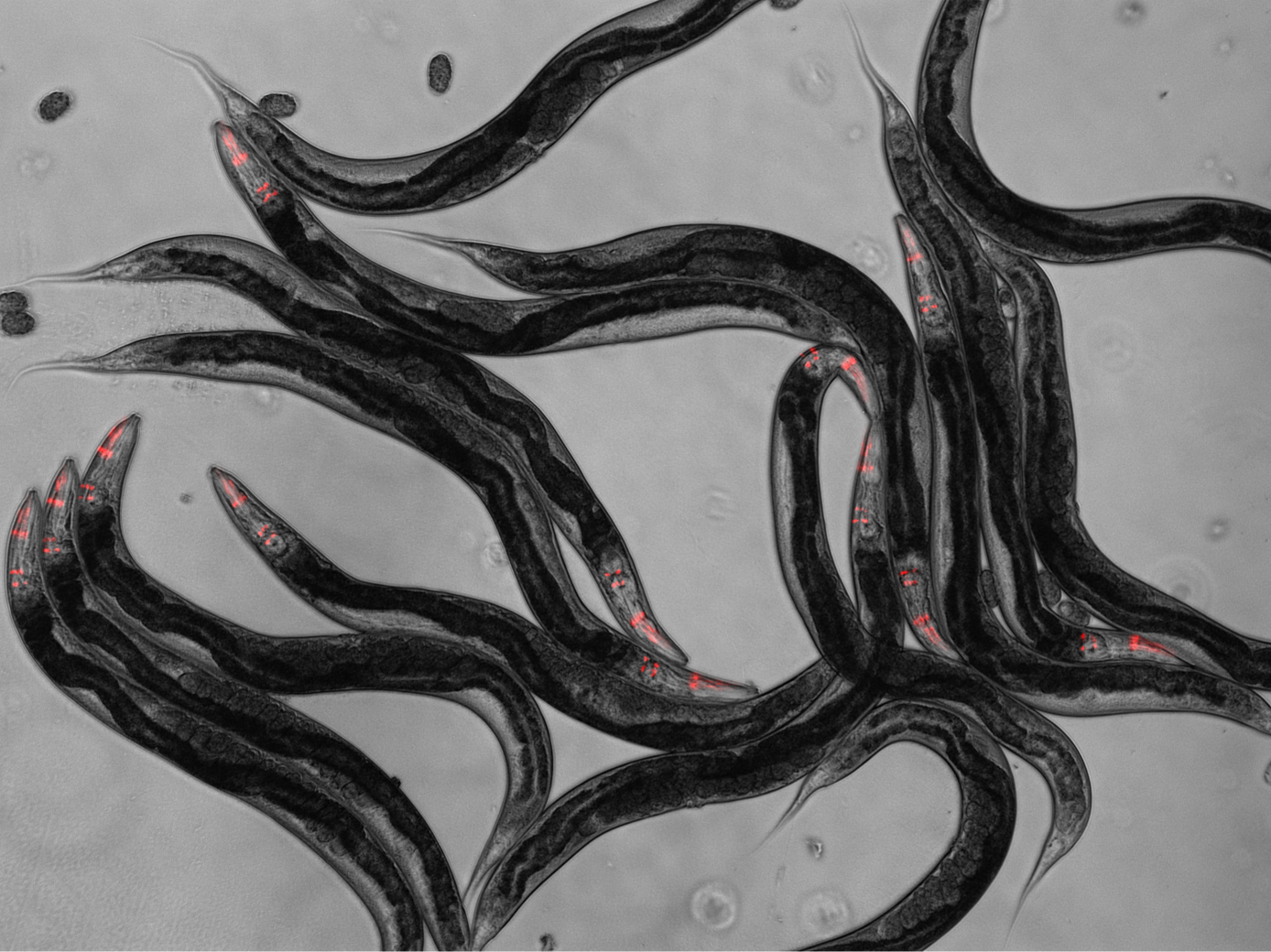

Supplement: Supplementary file 10 — Source data Fig. 7 [file 44319_2025_589_MOESM10_ESM.zip › EMBOR-2024-60913V2_Source-Data For Figure 7/7A/Day 1 control.tif]

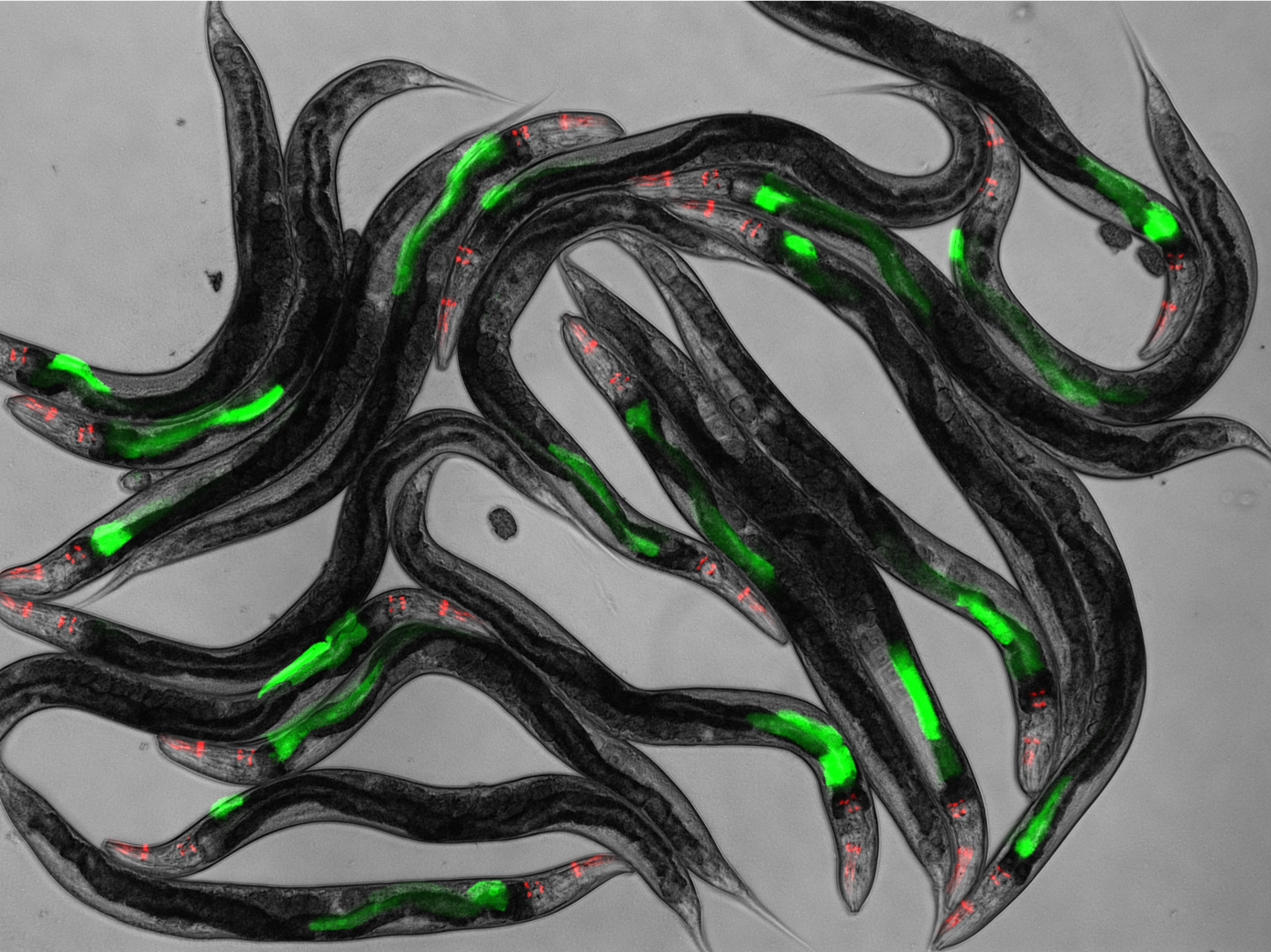

Supplement: Supplementary file 10 — Source data Fig. 7 [file 44319_2025_589_MOESM10_ESM.zip › EMBOR-2024-60913V2_Source-Data For Figure 7/7A/Day 1 Orsay.tif]

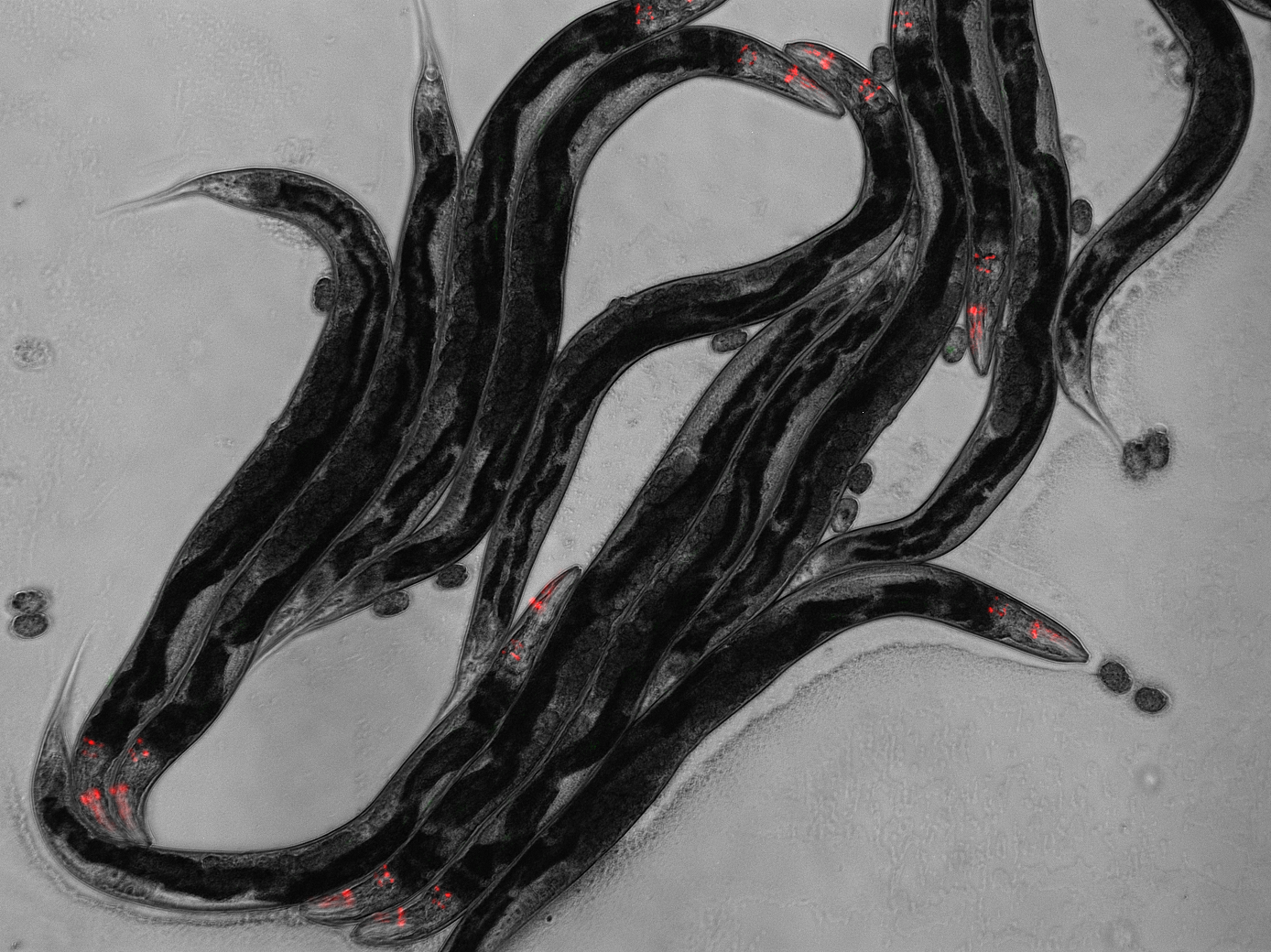

Supplement: Supplementary file 10 — Source data Fig. 7 [file 44319_2025_589_MOESM10_ESM.zip › EMBOR-2024-60913V2_Source-Data For Figure 7/7A/Day 4 control.tif]

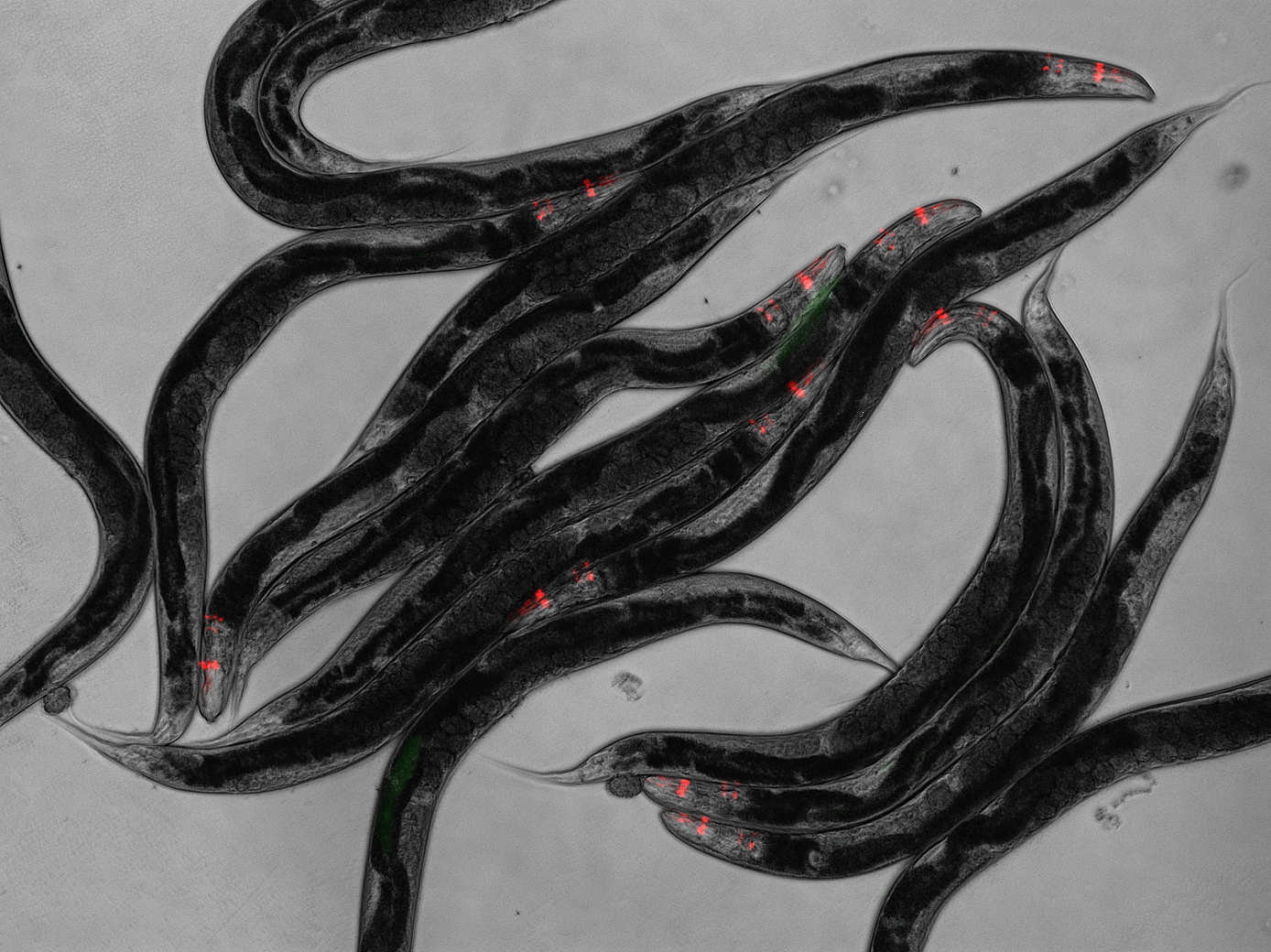

Supplement: Supplementary file 10 — Source data Fig. 7 [file 44319_2025_589_MOESM10_ESM.zip › EMBOR-2024-60913V2_Source-Data For Figure 7/7A/Day 4 Orsay.tif]

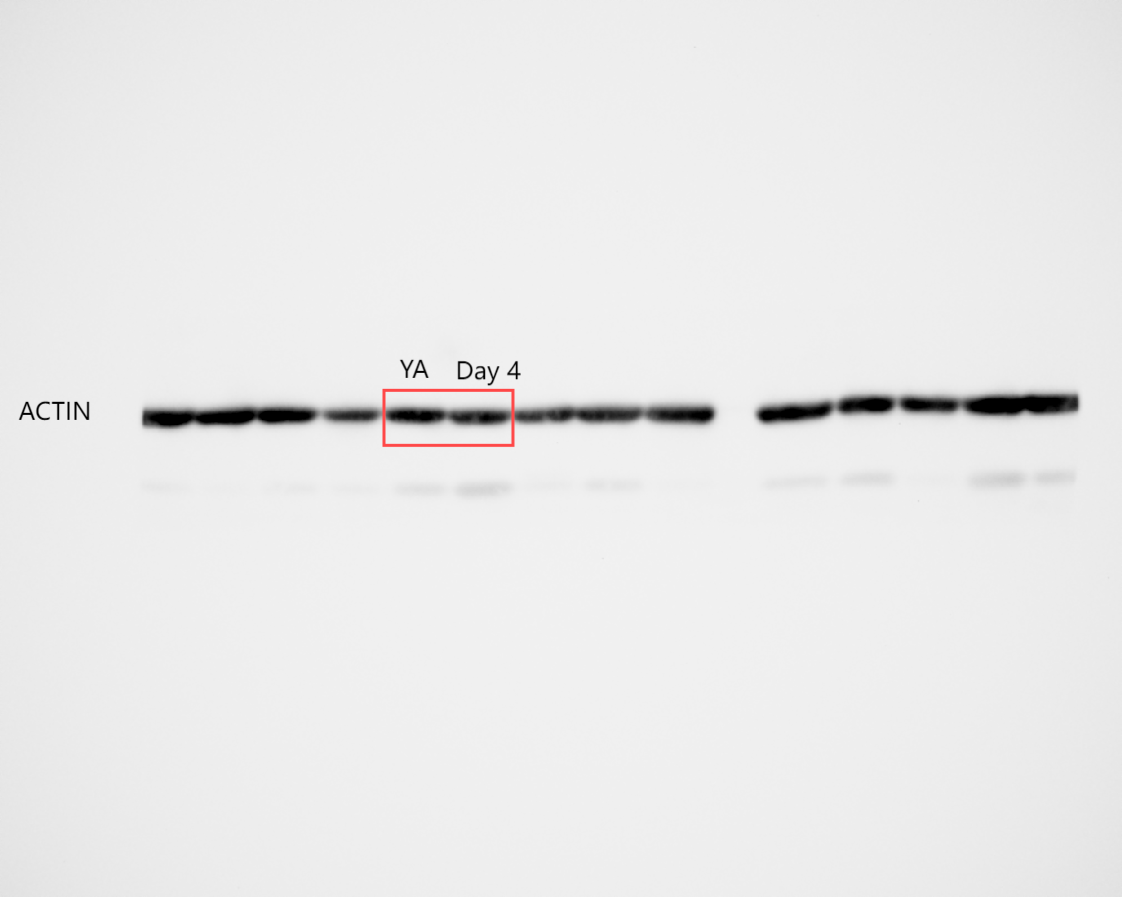

Supplement: Supplementary file 10 — Source data Fig. 7 [file 44319_2025_589_MOESM10_ESM.zip › EMBOR-2024-60913V2_Source-Data For Figure 7/7C/ACTIN.png]

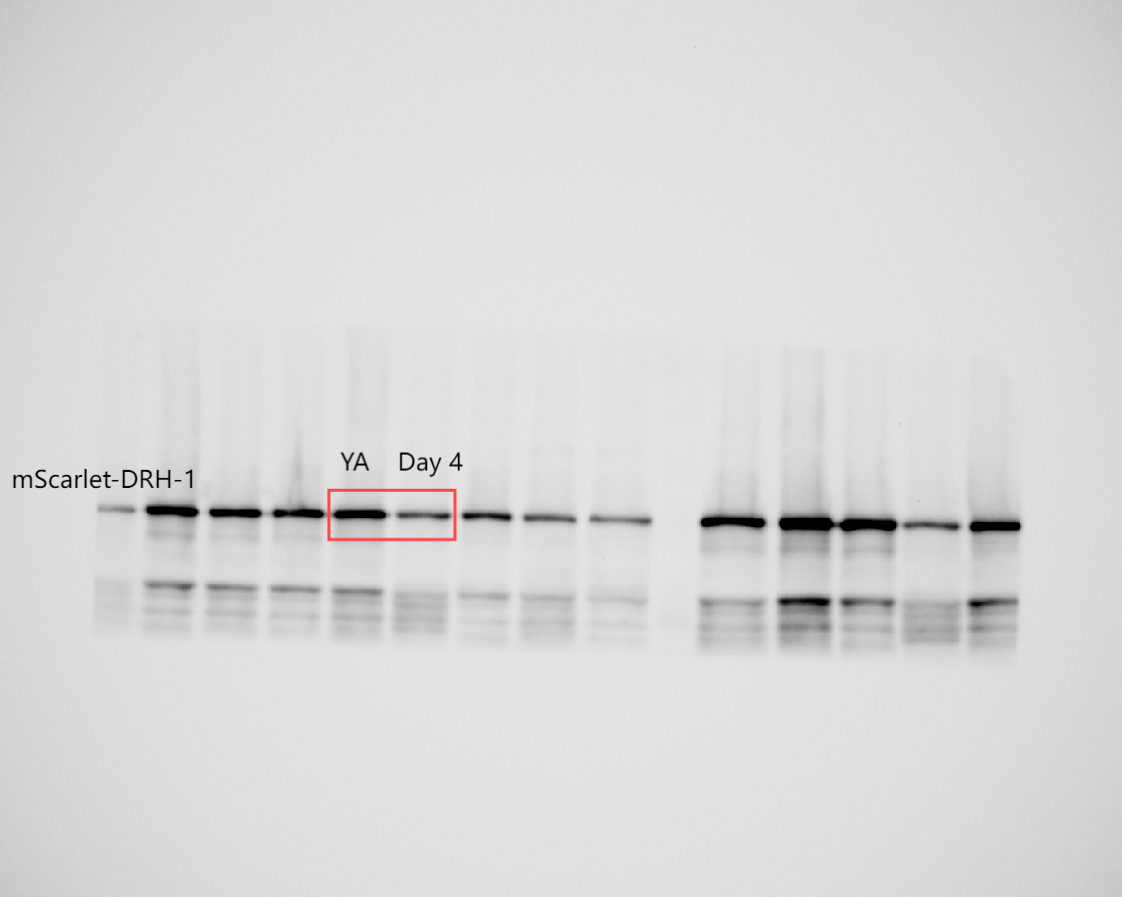

Supplement: Supplementary file 10 — Source data Fig. 7 [file 44319_2025_589_MOESM10_ESM.zip › EMBOR-2024-60913V2_Source-Data For Figure 7/7C/mScarlet-DRH-1.png]

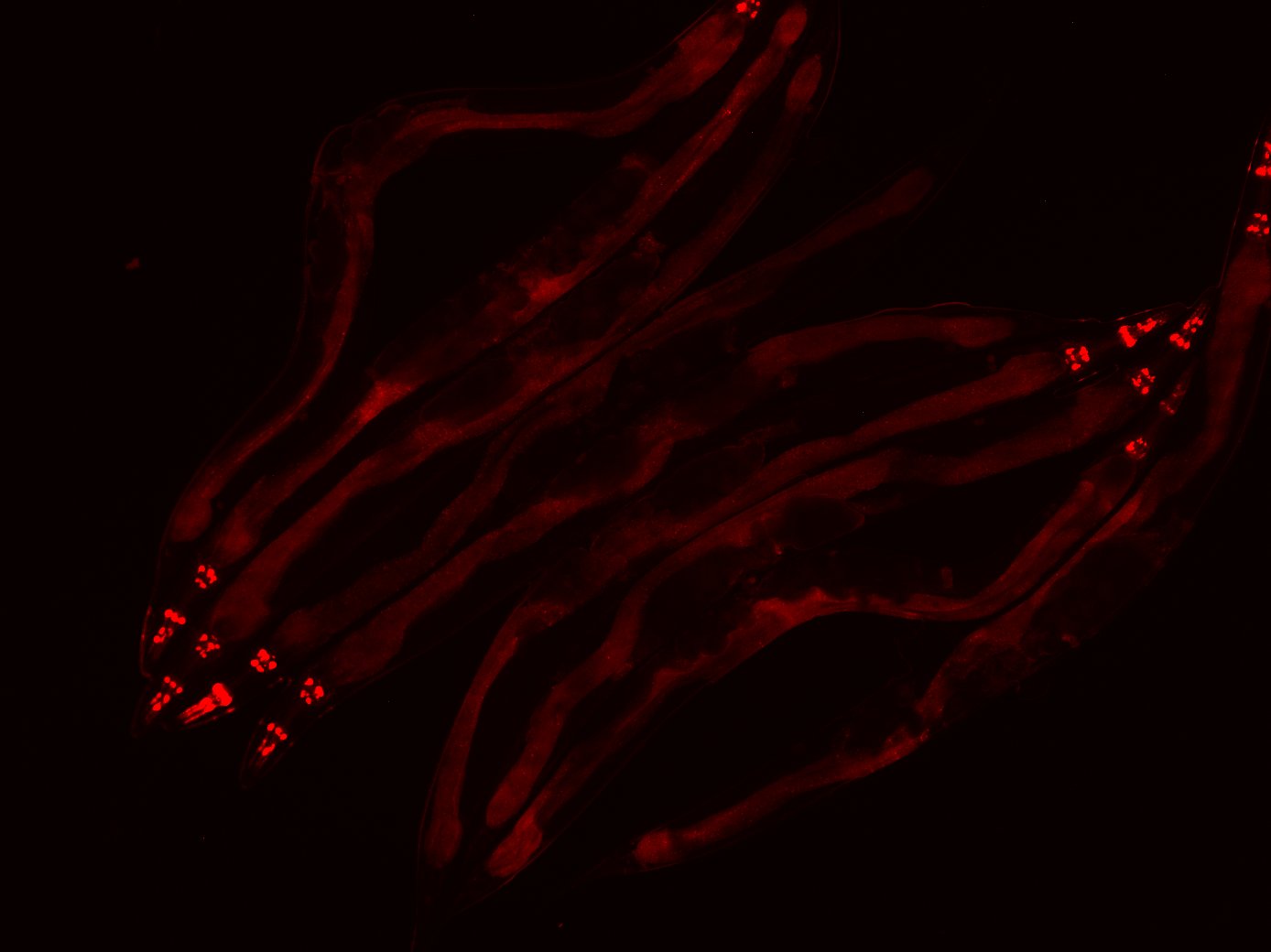

Supplement: Supplementary file 10 — Source data Fig. 7 [file 44319_2025_589_MOESM10_ESM.zip › EMBOR-2024-60913V2_Source-Data For Figure 7/7G/mScarlet-DRH-1 day4.jpg]

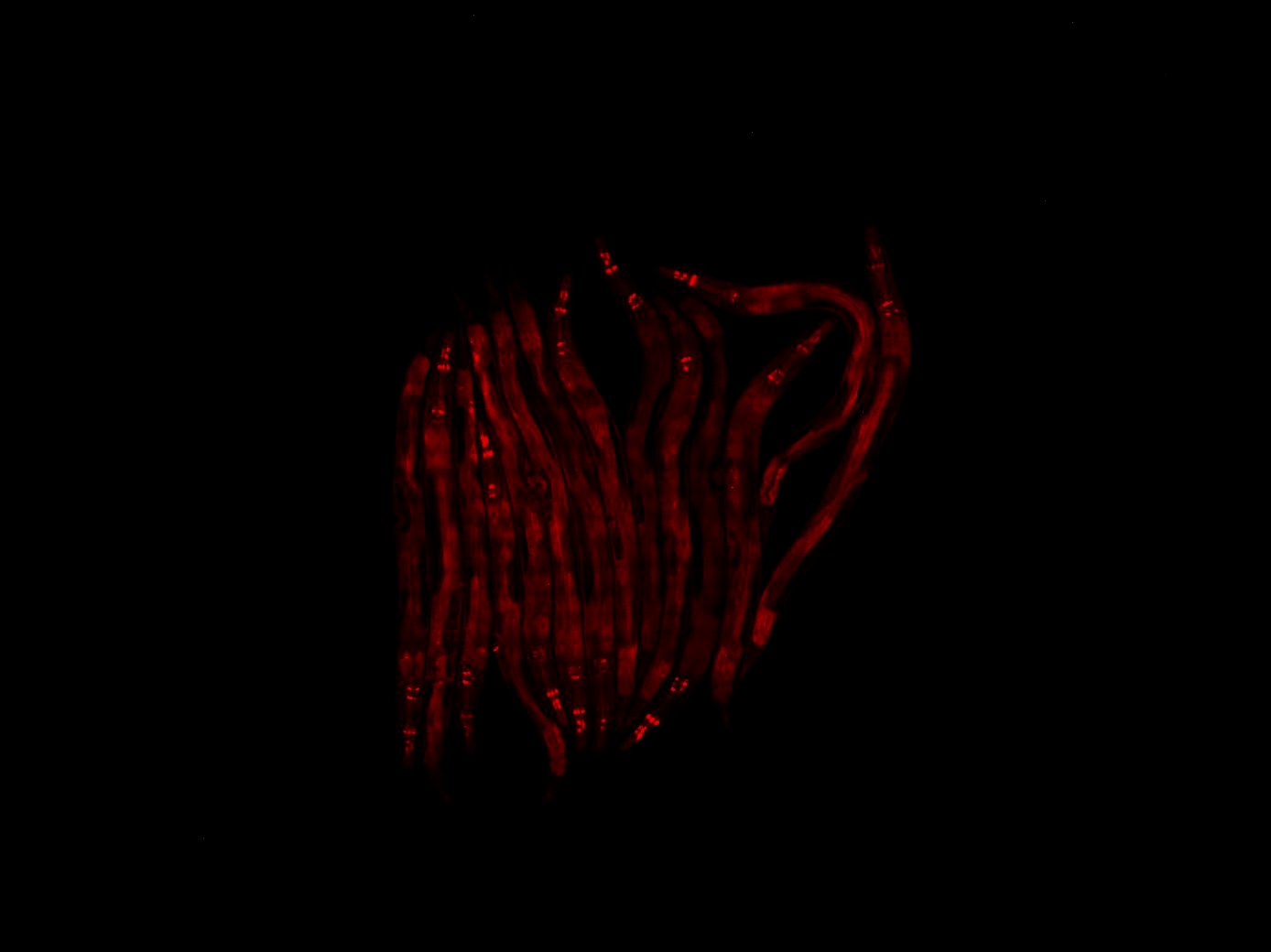

Supplement: Supplementary file 10 — Source data Fig. 7 [file 44319_2025_589_MOESM10_ESM.zip › EMBOR-2024-60913V2_Source-Data For Figure 7/7G/mScarlet-DRH-1 L4.jpg]

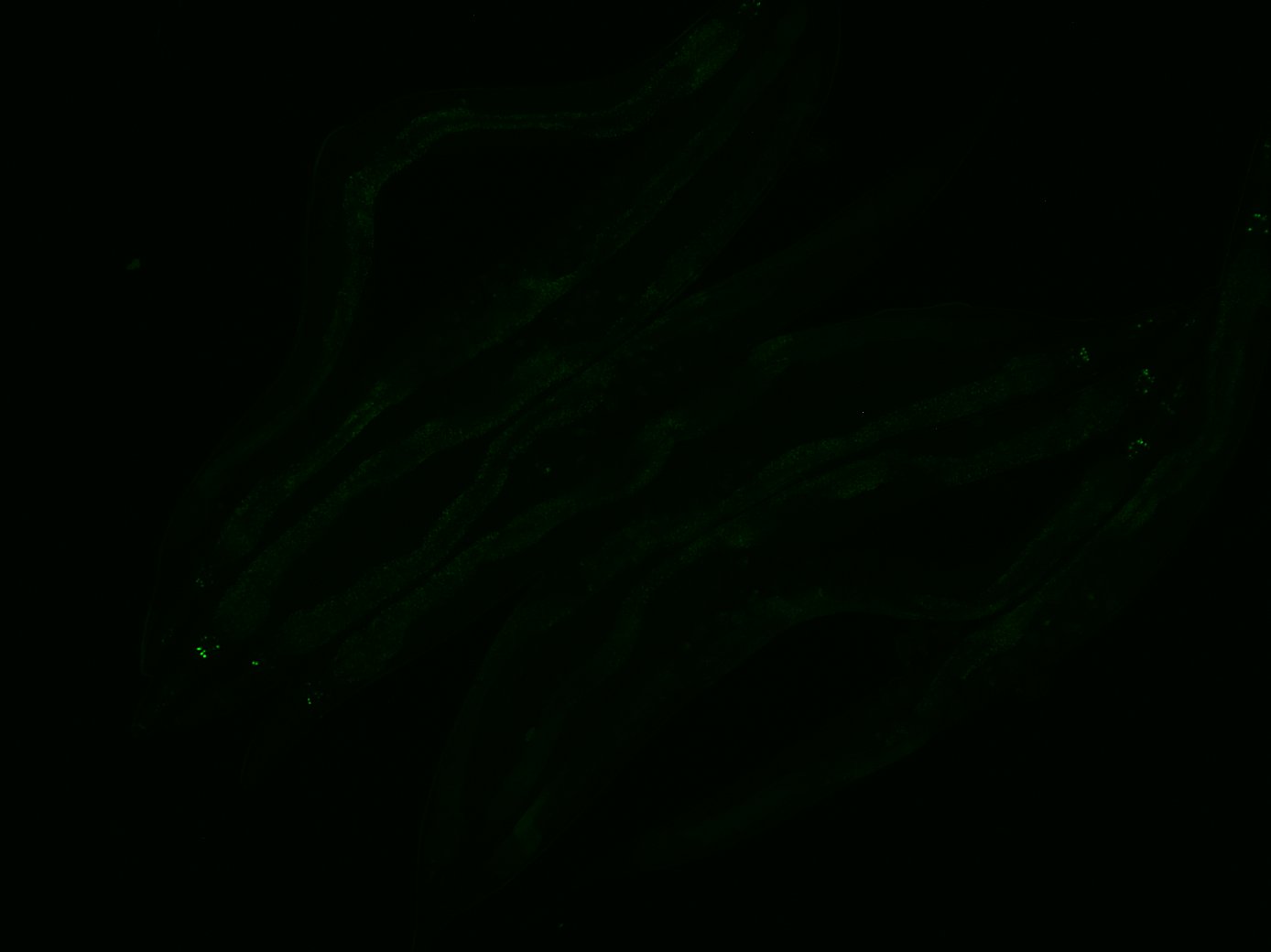

Supplement: Supplementary file 10 — Source data Fig. 7 [file 44319_2025_589_MOESM10_ESM.zip › EMBOR-2024-60913V2_Source-Data For Figure 7/7G/mScarlet-DRH-1 OE induced pals-5-gfp day4.jpg]

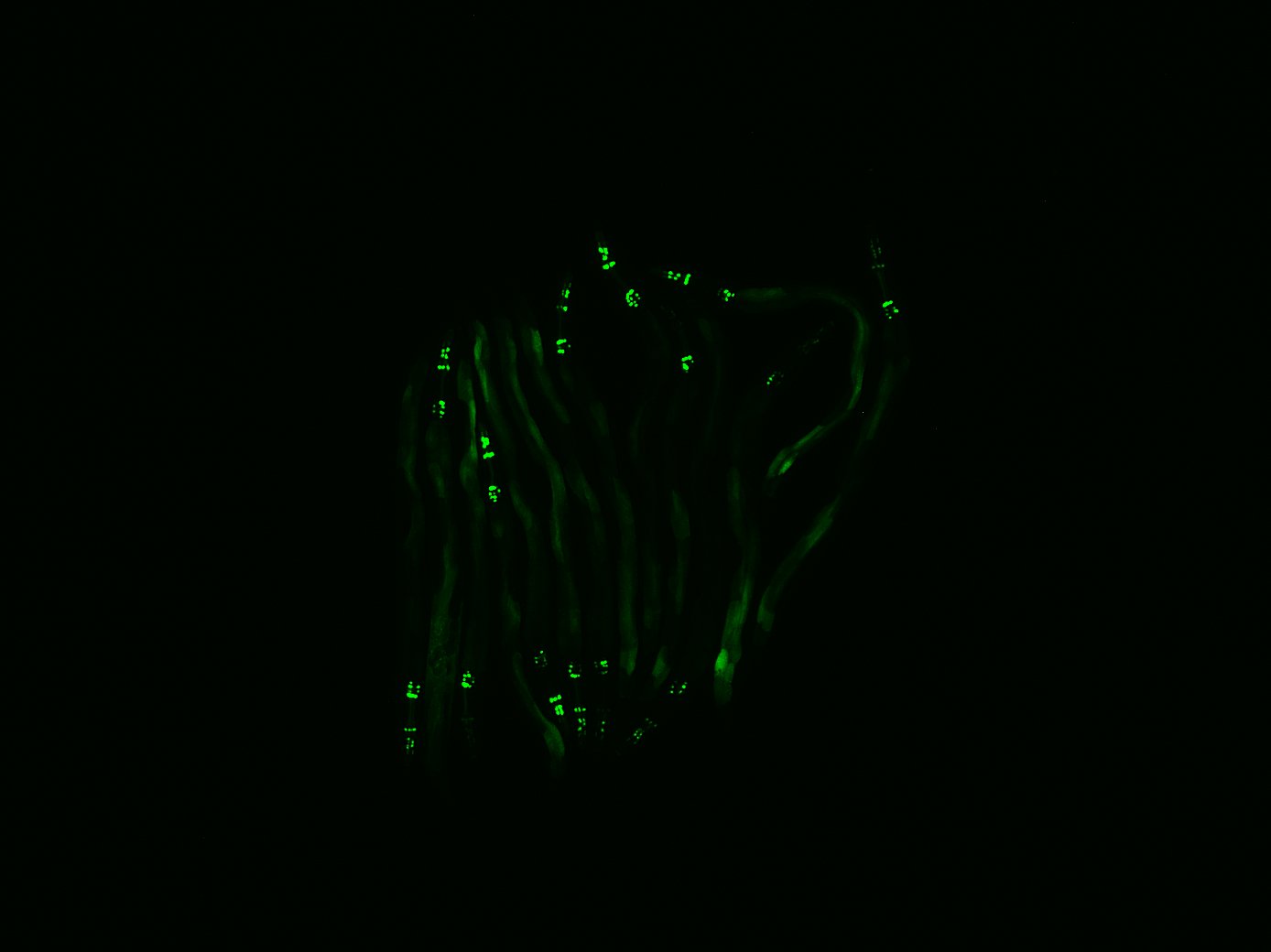

Supplement: Supplementary file 10 — Source data Fig. 7 [file 44319_2025_589_MOESM10_ESM.zip › EMBOR-2024-60913V2_Source-Data For Figure 7/7G/mScarlet-DRH-1OE induced pals-5-gfp L4.jpg]

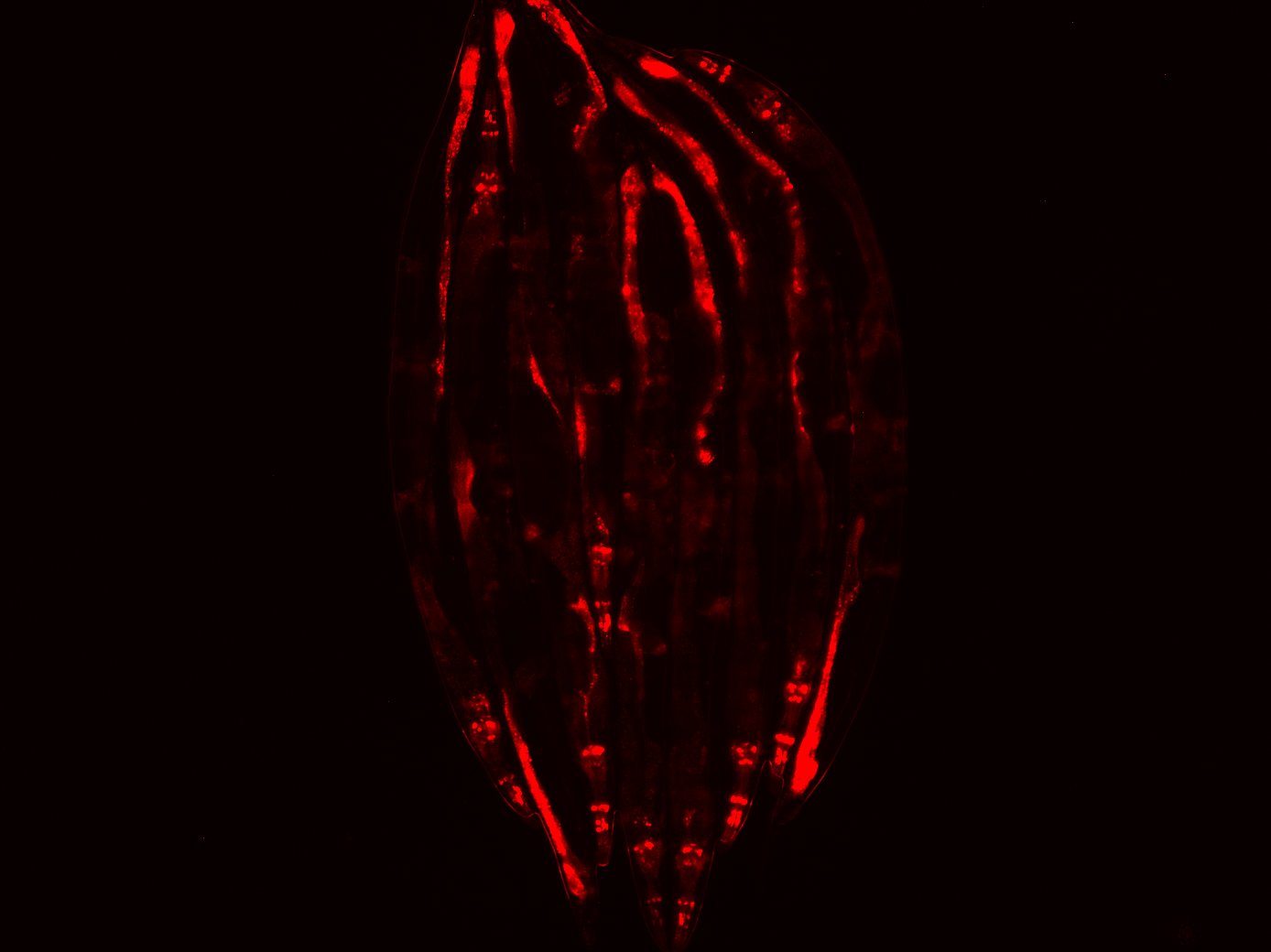

Supplement: Supplementary file 10 — Source data Fig. 7 [file 44319_2025_589_MOESM10_ESM.zip › EMBOR-2024-60913V2_Source-Data For Figure 7/7H/mScarlet-DRH-1(2KR) day4.jpg]

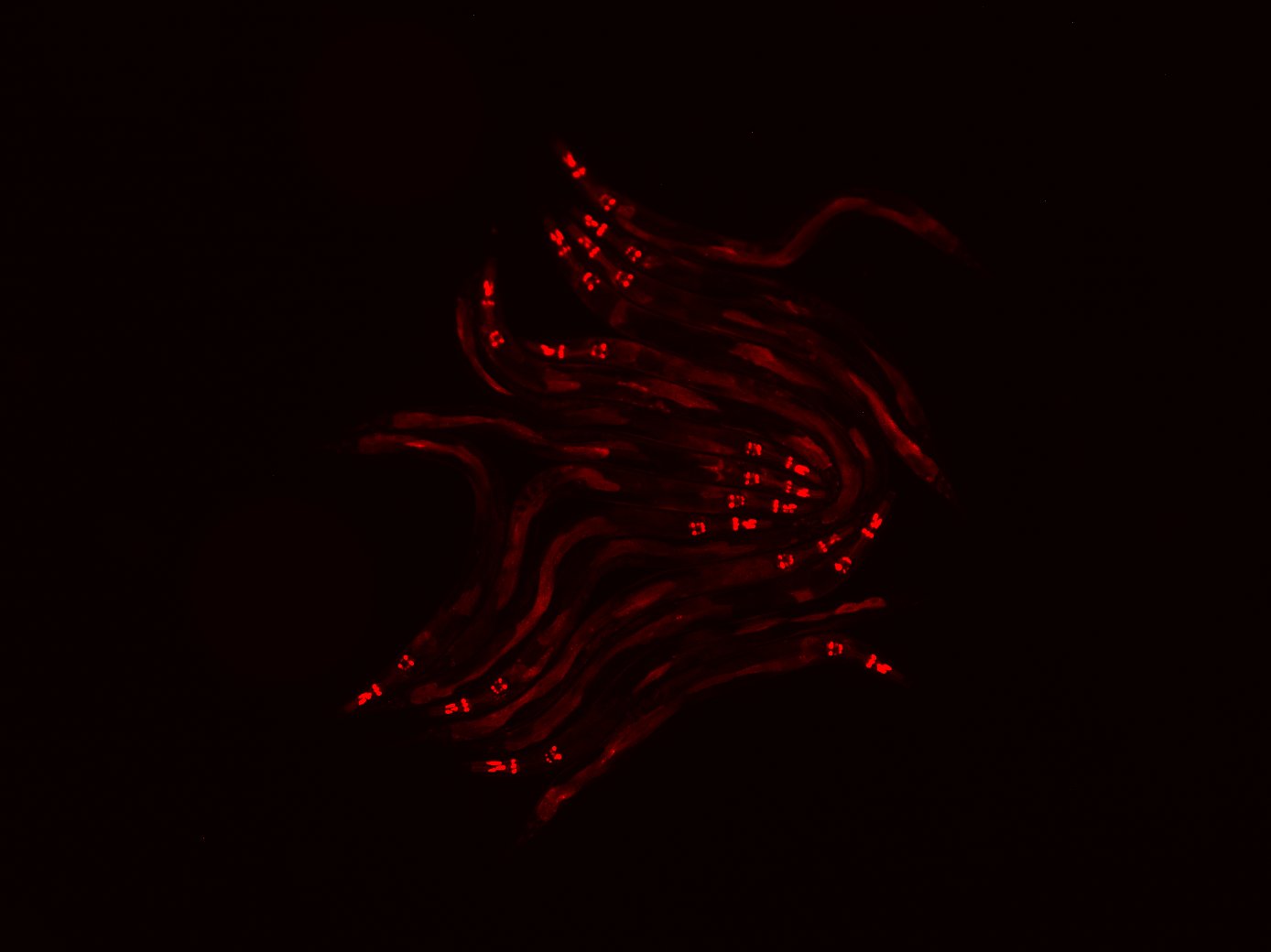

Supplement: Supplementary file 10 — Source data Fig. 7 [file 44319_2025_589_MOESM10_ESM.zip › EMBOR-2024-60913V2_Source-Data For Figure 7/7H/mScarlet-DRH-1(2KR) L4.jpg]

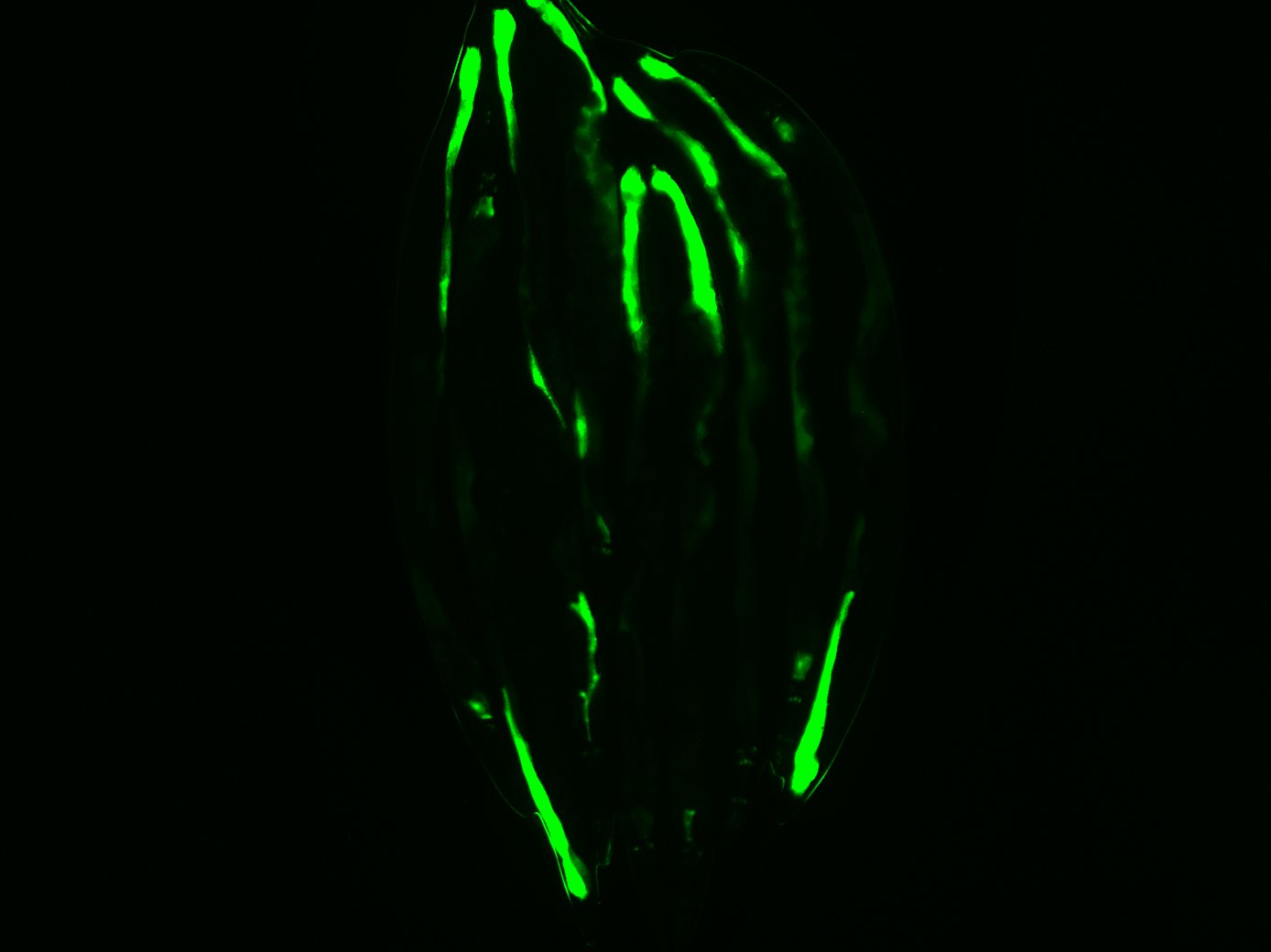

Supplement: Supplementary file 10 — Source data Fig. 7 [file 44319_2025_589_MOESM10_ESM.zip › EMBOR-2024-60913V2_Source-Data For Figure 7/7H/mScarlet-DRH-1(2KR) OE induced pals-5-gfp day4.jpg]

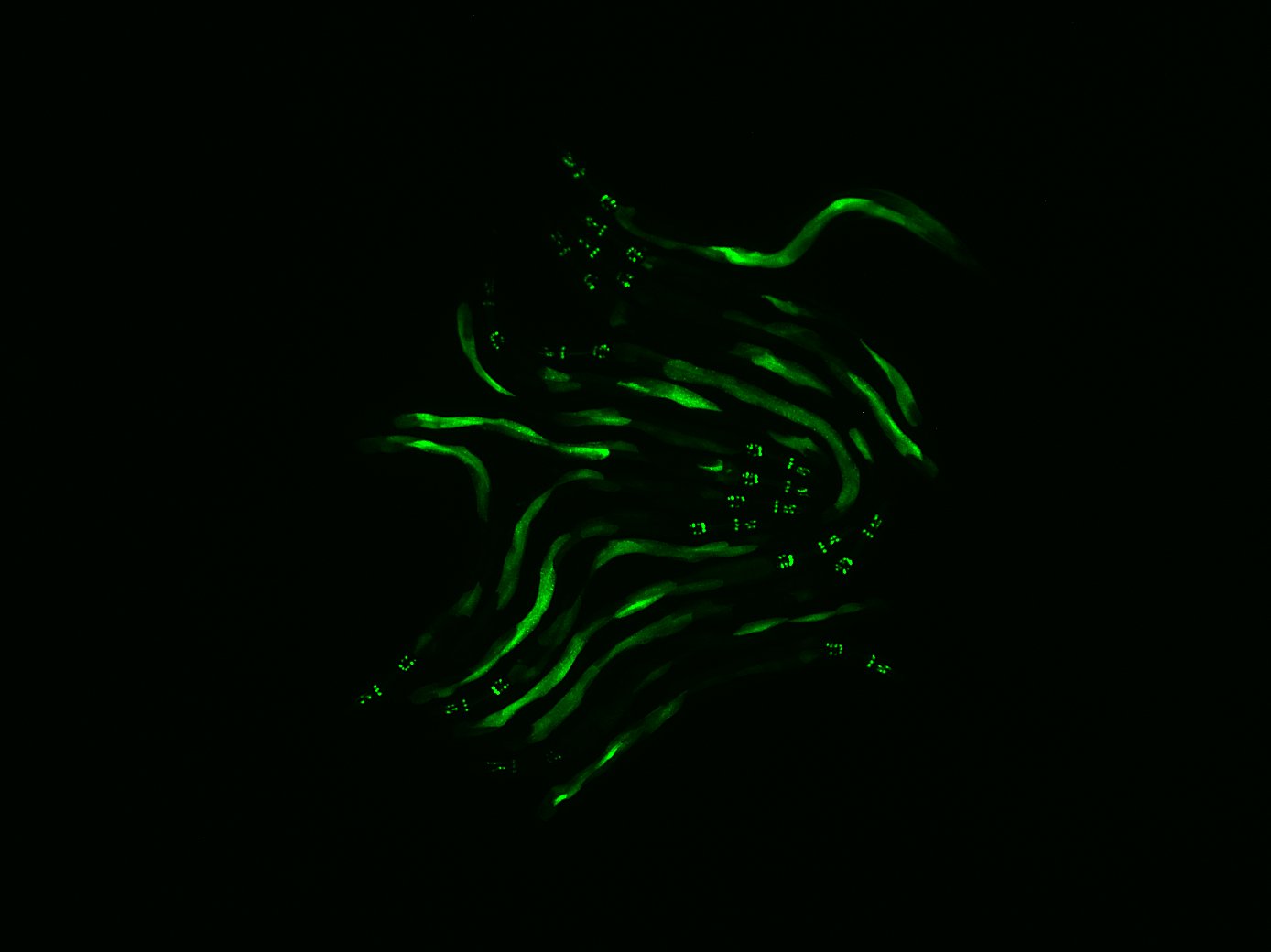

Supplement: Supplementary file 10 — Source data Fig. 7 [file 44319_2025_589_MOESM10_ESM.zip › EMBOR-2024-60913V2_Source-Data For Figure 7/7H/mScarlet-DRH-1(2KR) OE induced pals-5-gfp L4.jpg]
