# Supplementary material for: Aging impairs the antiviral defense in Caenorhabditis elegans due to loss of DRH-1/RIG-I deSUMOylation by ULP-4/SENP7
Source: EMBO Rep. 2025 Oct 2;26(22):5459–82. doi: 10.1038/s44319-025-00589-0 (PMC12635358; doi:10.1038/s44319-025-00589-0)
Supplement: Supplementary file 11 — Expanded View Figures [file 44319_2025_589_MOESM11_ESM.pdf]

Expanded View Figures

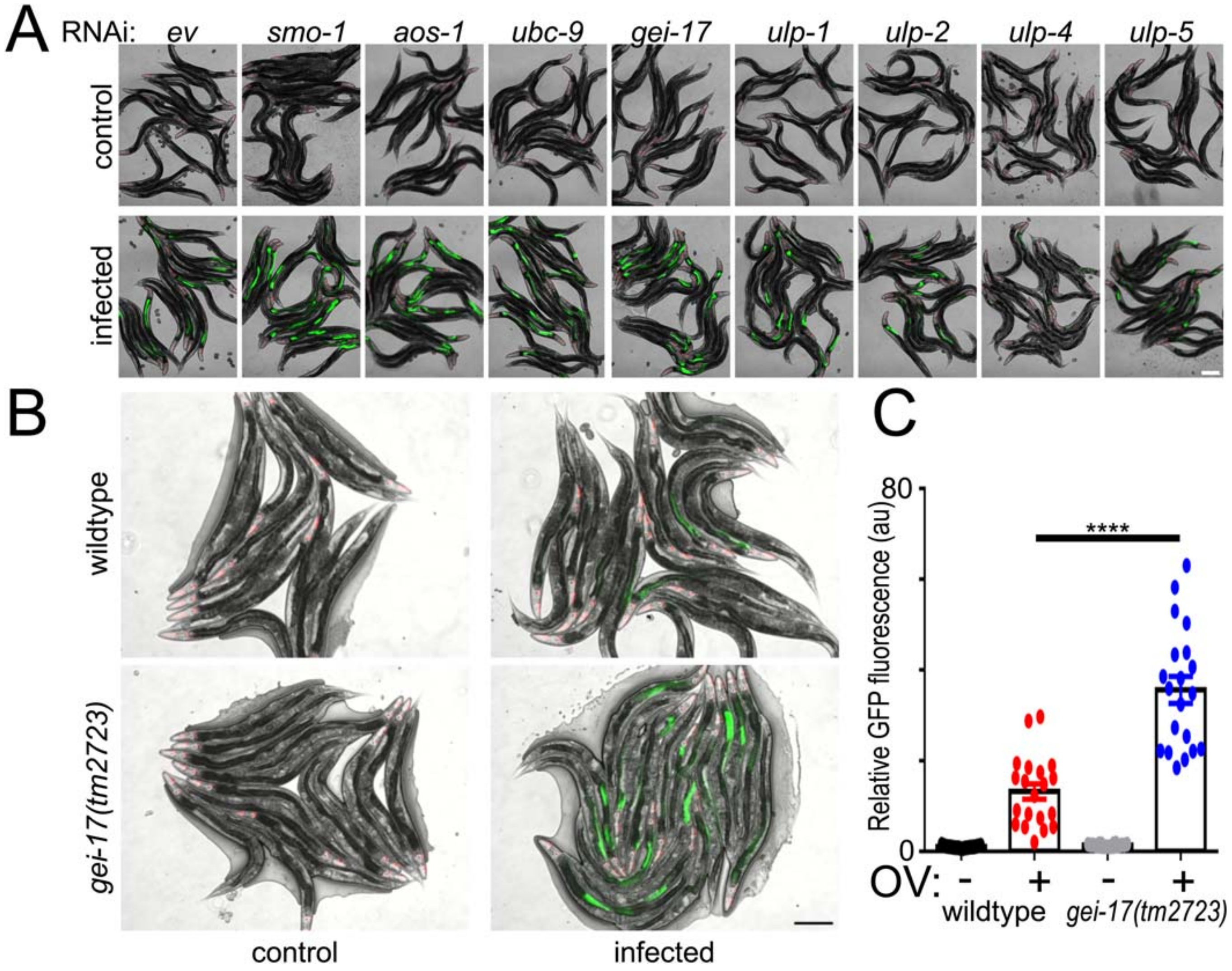

Figure EV1. Inhibition of SUMOylation amplifies IPR induction after viral infection.

(**A**) Representative images of *pals-5p::GFP* expression after RNAi of the indicated component of the SUMOylation machinery +/− viral infection. Quantification of GFP fluorescence is in Fig. 1B. Images of the empty vector (ev) and *ulp-4(RNAi)* are also shown in Fig. 1A. (**B**) Representative images of *pals-5p::GFP* expression in infected wildtype and *gei-17(tm2723)* null mutant animals. Scale bar = 200 µm. (**C**) Quantification of GFP fluorescence intensity of samples in (**B**). ****$P < 0.0001$. In all cases, values are the mean of 20 animals per trial, across three independent biological trials; error bars represent the SEM. A two-tailed *t*-test was used to calculate *P* value.

 © The Author(s)

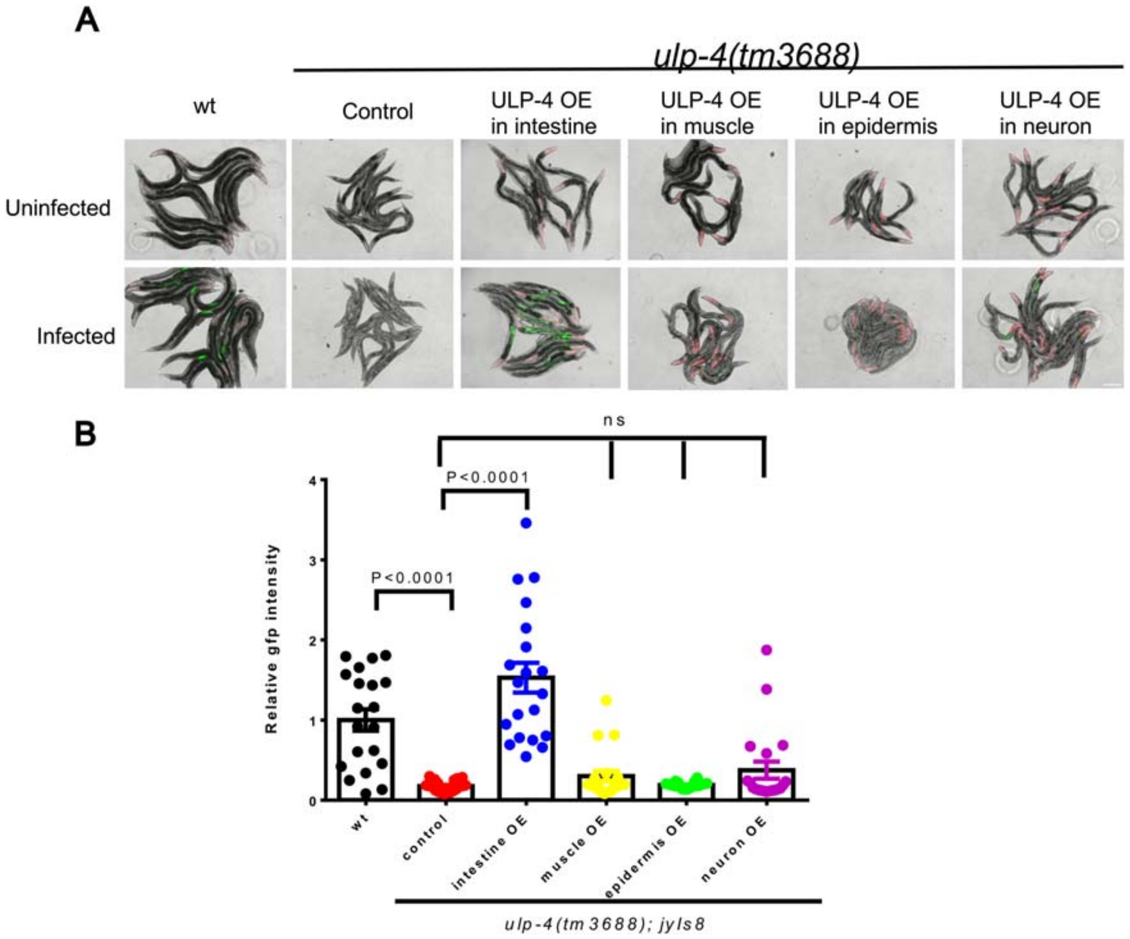

Figure EV2. ULP-4 does not act outside of the intestine to regulate IPR following viral infection.

(**A**) Representative images of *pals-5p::GFP* expression +/- viral infection of animals that were: wildtype, *ulp-4(tm3688)*, *ulp-4(tm3688);artEx95(ges-1p::ULP-4)*, *ulp-4(tm3688);artEx96(myo-3p::ULP-4)*, *ulp-4(tm3688);artEx104(dpy-7p::ULP-4)*, or *ulp-4(tm3688);artEx103(rab-3p::ULP-4)*, which restored ULP-4 expression within the intestine, body wall muscle, epidermis, or nervous system, respectively. Scale bar = 200 µm. (**B**) RT-qPCR analysis of endogenous *pals-5* mRNA of samples in (**A**). *P* values are provided within the panel. In all cases, values are the mean of 20 animals per trial, across three independent biological trials; error bars are SEM. A two-tailed *t*-test was used to calculate *P* values.

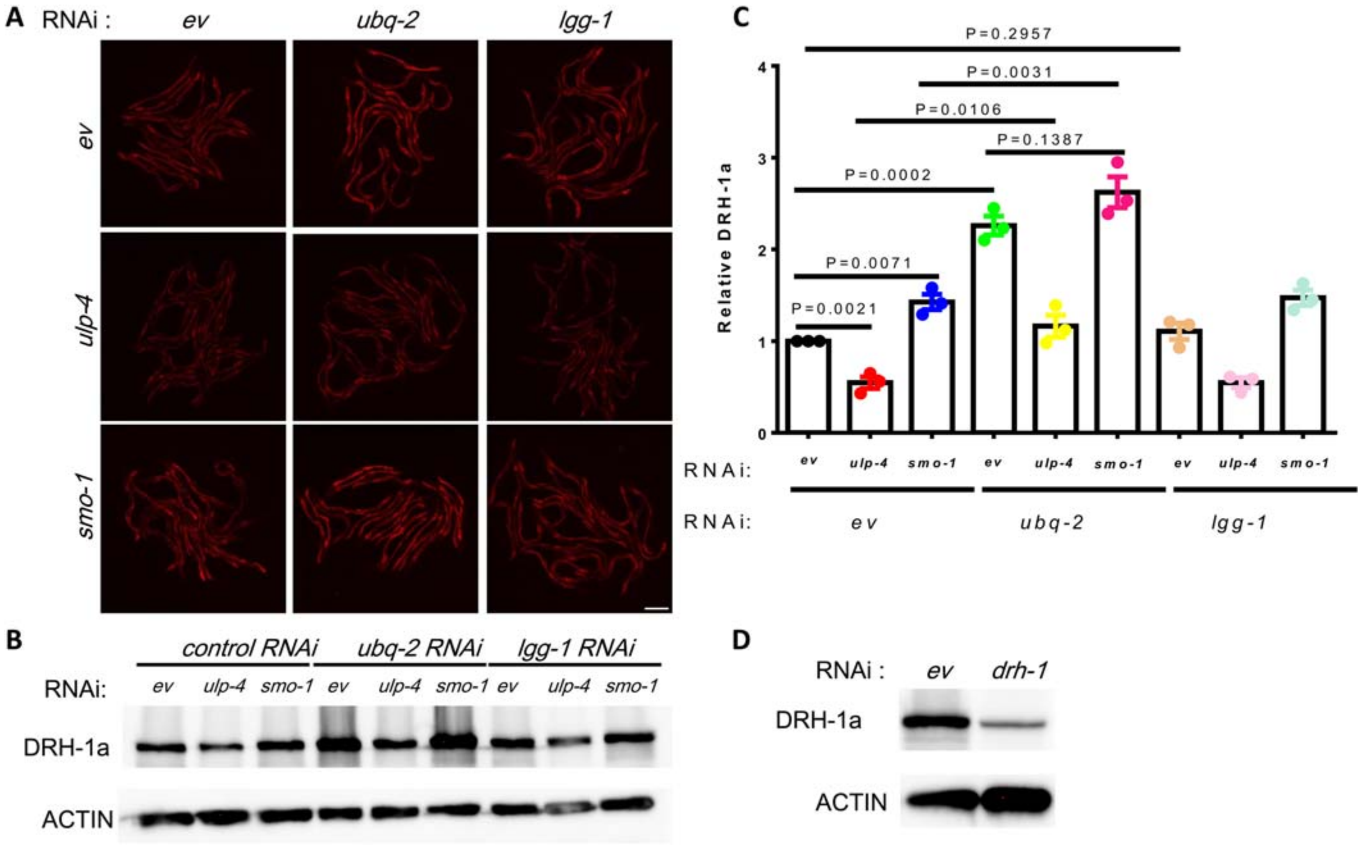

Figure EV3. DRH-1 is degraded through proteasome.

(**A**) Representative images of *mScarlet::DRH-1* expression after RNAi treatment with: empty vector, *ubq-2*, *lgg-1*, *ulp-4*, *ulp-4*+*ubq-2*, *ulp-4*+*lgg-1*, *smo-1*, *smo-1*+*ubq-2*, or *smo-1*+*lgg-1*, respectively. Scale bar = 200 μm. (**B**) Representative immunoblot of DRH-1 levels. (**C**) Quantification of DRH-1 protein levels. *P* values are provided within the panel. (**D**) Representative immunoblot of DRH-1 levels after empty vector or *drh-1(RNAi)*. In all cases, values are the mean of 20 animals per trial, across three independent biological trials; error bars are SEM. A two-tailed *t*-test was used to calculate *P* values.

 © The Author(s)

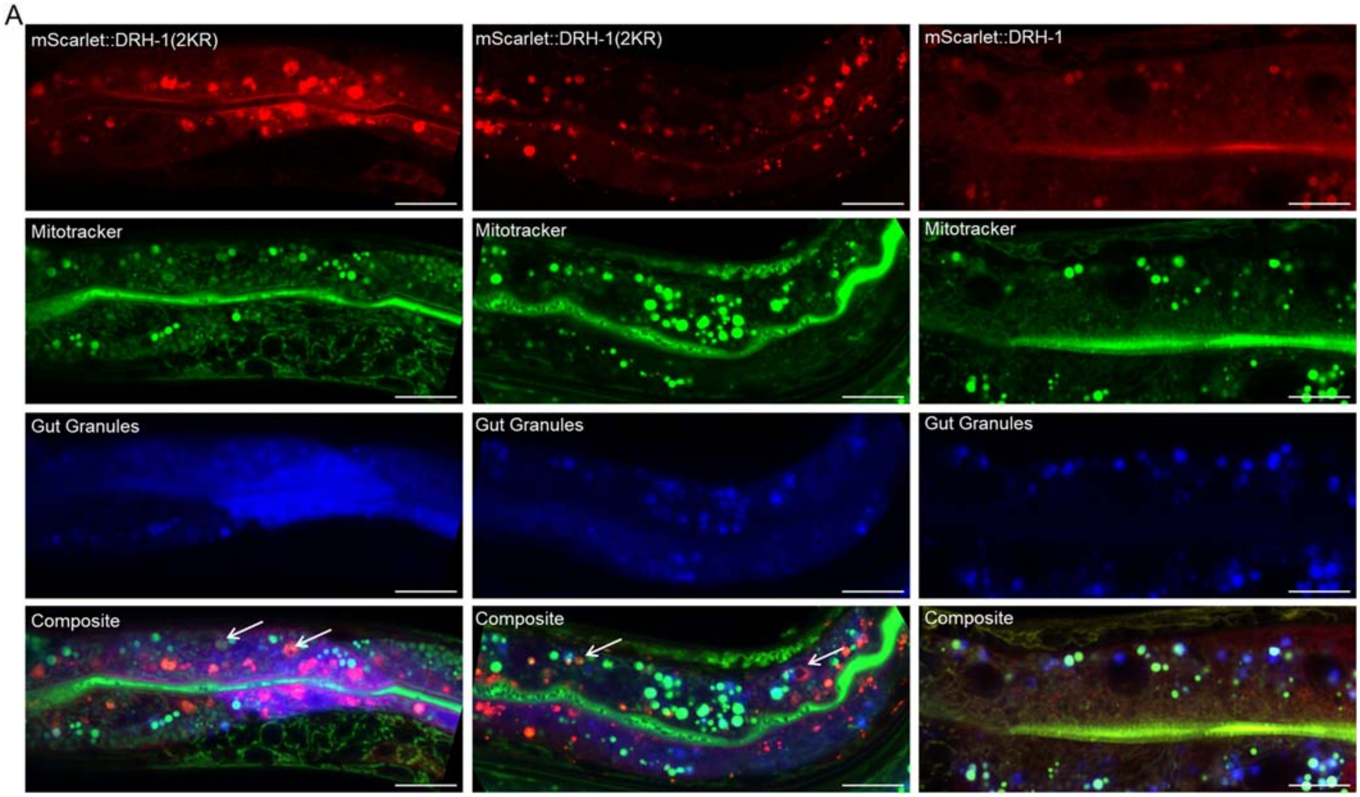

Figure EV4. NonSUMOylated DRH-1 translocates to the mitochondria.

(**A**) Representative images of an intestinal cell of an animal expressing either *mScarlet::DRH-1 or mScarlet::DRH-1(2KR)*. Far Red Mitotracker was used to mark mitochondria, lysosomal-related organelles/gut granules are indicated (405-nm blue channel). Arrows highlight NonSUMOylated DRH-1 localization to the mitochondria periphery. Scale bar = 10 μm.

© The Author(s)

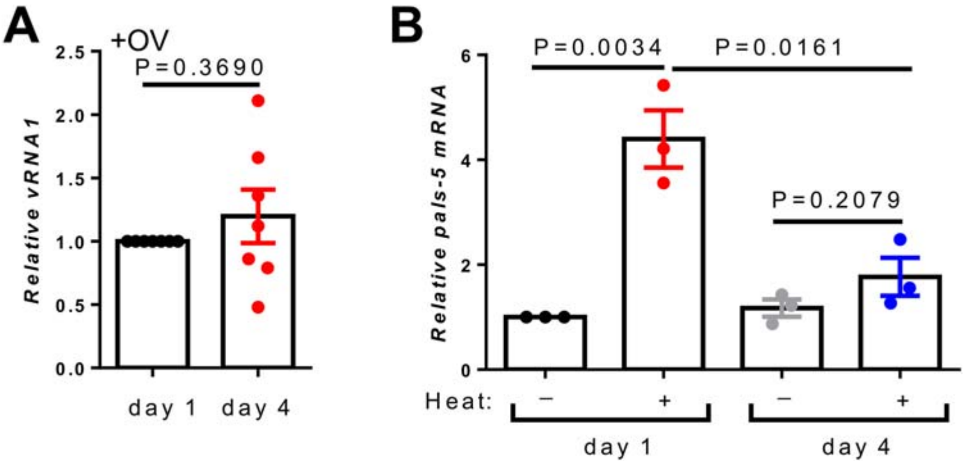

Figure EV5. The IPR induced by a non-viral trigger declines during aging.

(**A**) RT-qPCR analysis of viral *RNA1* levels in day 1 or day 4 animals. Viral infection was initiated at L4 or day 3 of adulthood, and animals were collected 24 h later. (**B**) RT-qPCR analysis of endogenous *pals-5* induction in day 1 or day 4 animals treated with prolonged heat stress. Heat stress was initiated at L4 or day 3 of adulthood at 28 °C for 24 h. Control plates were maintained at 20 °C. In all cases, values are the mean across three independent biological trials; error bars are SEM. A two-tailed *t*-test was used to calculate *P* values, which are provided within the panels.

 © The Author(s)
